# Gp93 safeguards tissue homeostasis by preventing ROS-JNK-mediated apoptosis

**DOI:** 10.1016/j.redox.2025.103537

**Published:** 2025-02-08

**Authors:** Meng Xu, Wanzhen Li, Ruihong Xu, Lixia Liu, Zhihan Wu, Wenzhe Li, Chao Ma, Lei Xue

**Affiliations:** a Department of Nuclear Medicine, Shanghai 10th People's Hospital, Shanghai Key Laboratory of Signaling and Diseases Research, School of Life Science and Technology, Tongji University, Shanghai, China; b National Clinical Research Center for Interventional Medicine, Shanghai 10th People's Hospital, 200072, Shanghai, China

**Keywords:** *Drosophila*, ROS, Gp93, JNK signaling, Apoptosis, HSP90B1

## Abstract

Reactive oxygen species (ROS) play a pivotal role in maintaining tissue homeostasis, yet their overabundance can impair normal cellular functions, induce cell death, and potentially lead to neurodegenerative disorders. This study identifies *Drosophila* Glycoprotein 93 (Gp93) as a crucial factor that safeguards tissue homeostasis and preserves normal neuronal functions by preventing ROS-induced, JNK-dependent apoptotic cell death. Firstly, loss of *Gp93* induces JNK-dependent apoptosis primarily through the induction of ROS. Secondary, neuro-specific depletion of *Gp93* results in ROS-JNK-mediated neurodegeneration. Thirdly, overexpression of Gp93 effectively curtails oxidative stress and neurodegeneration caused by paraquat exposure or the aging process. Furthermore, these functions of Gp93 can be substituted by its human ortholog, HSP90B1. Lastly, depletion of *HSP90B1* in cultured human cells triggers ROS production, JNK activation, and apoptosis. Thus, this study not only unveils a novel physiological function of Gp93, but also provides valuable insights for understanding the physiological and pathological functions of human HSP90B1.

## Highlights

• Loss of *Gp93* induces JNK-dependent apoptosis primarily through the induction of ROS.
• Neuro-specific depletion of *Gp93* results in ROS-JNK-mediated neurodegeneration.
• Overexpression of Gp93 effectively curtails oxidative stress and neurodegeneration.
• Human Hsp90B1 retains Gp93's function in preventing ROS-JNK signaling-mediated apoptosis.
• Depletion of *HSP90B1* in human cells triggers ROS production, JNK activation, and apoptosis.

## Graphical abstract

**Figure:**
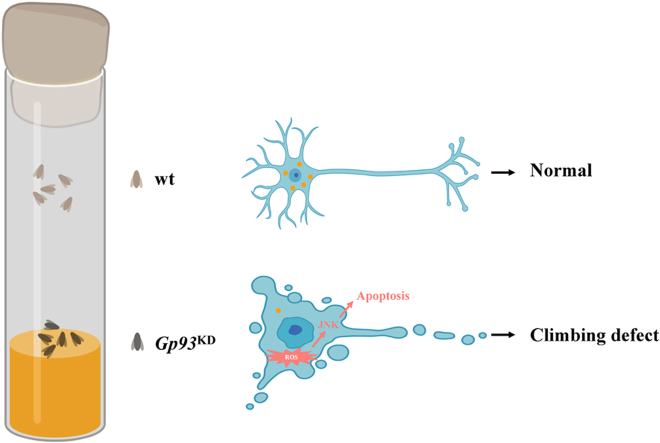

## 1 Introduction

Reactive oxygen species (ROS) are byproducts of cellular aerobic metabolism and are tightly regulated within cellular systems [1]. They serve as important signaling molecules that influence cell development and play a crucial role in maintaining tissue homeostasis [2]. ROS facilitate post-translational modifications of a variety of enzymes and transcription factors, thereby modulating their biological activity and interacting partners, which, in turn, impacts cellular processes such as proliferation, survival, differentiation, and metabolism [[3], [4], [5]]. Modulate levels of ROS are beneficial for organisms, aiding in pathogen defense, wound healing, and tissue repair. However, an excessive accumulation of ROS can damage cell structures (lipids and membranes), proteins, and nucleic acids, contributing to the development of cancer, cardiovascular disease, and neurodegenerative disorders [6]. To counterbalance ROS, cells employ an enzymatic antioxidant defense system, including enzymes like superoxide dismutase (Sod), catalase (Cat), and glutathione peroxidase (Gtpx), to maintain redox homeostasis [7]. The c-Jun N-terminal kinase (JNK) signaling pathway is a primary response to ROS [8,9].

In mammals, molecules linking ROS and JNK activity have been reported [3]. Two key enzymes, apoptosis signal-regulating kinase 1 (Ask1) and glutathione-S-transferase P (GSTP), have been found to independently modulate JNK activity through distinct mechanisms. Under physiological conditions, thioredoxin acts as an inhibitor of Ask1, a member of the mitogen-activated protein kinase kinase kinase (MAPKKK) family. However, in the presence of ROS, the oxidation of critical cysteine residues in thioredoxin leads to its dissociation from Ask1, which in turn phosphorylates MAPK kinase 4/7 and activates JNK [10]. GSTP typically functions as an inhibitor of JNK. During oxidative stress, its dissociation from JNK permits phosphorylation and activation of the kinase [11]. In *Drosophila*, neuronal JNK responds to ROS to shape neuronal morphology [8].

*Drosophila*, which expresses a single JNK protein encoded by *basket* (*bsk*), unlike mammals with three JNK proteins (JNK1/2/3), offers a valuable model for studying JNK signaling [12]. Bsk is activated through phosphorylation by JNK kinases (JNKKs), which are in turn phosphorylated by JNK kinases kinases (JNKKKs) [13]. Phosphorylated Bsk activates the transcription factor AP-1, composed of c-Jun and Fos, thereby promoting the expression of target genes [14]. Additionally, Bsk can be dephosphorylated by the protein product of *puckered* (*puc*) gene, a target of AP-1 and a negative feedback regulator of JNK signaling [15].

The *Drosophila* heat shock protein 90 (HSP90) family encompasses three key proteins: Hsp90, Trap1, and Glycoprotein 93 (Gp93). The absence of Hsp90 has been shown to inhibit the expression of the antimicrobial peptide Mtk and to prevent eye degeneration caused by polyglutamine aggregates [16]. Trap1, on the other hand, mitigates neurotoxicity induced by the human α-Synuclein protein [17] and addresses mitochondrial dysfunction, thereby rescuing neurodegeneration associated with Parkinson's disease (PD) [18]. Gp93, while known to regulate gut epithelial homeostasis and dendrite development [19,20], has not been fully explored in terms of its neural functions. In mammals, heat shock protein 90 beta family member 1 (HSP90B1), also known as Glycoprotein 96 (GP96), and homologous to Gp93, has been implicated in cancer progression [21] and cell protection against oxidative stress [22,23], yet its precise physiological function in development remains to be fully elucidated.

This study unveils a novel function of *Gp93* in *Drosophila,* as depletion of *Gp93* triggers oxidative stress, leading to JNK-dependent apoptosis and subsequent neurodegeneration. These functions of Gp93 are conserved by HSP90B1, as the introduction of HSP90B1 into *Drosophila* can rescue Gp93 depletion-induced ROS-JNK-mediated apoptosis, and the knockdown of *HSP90B1* in human cells results in ROS induction, JNK activation, and apoptosis. Thus, this research not only sheds light on a previously unknown physiological function of Gp93, but also provides valuable insights that may guide future research in mammals related to processes involving oxidative stress.

## 2 Results

### 2.1 Gp93 inhibits cell death in eye development

**Fig. 1:**
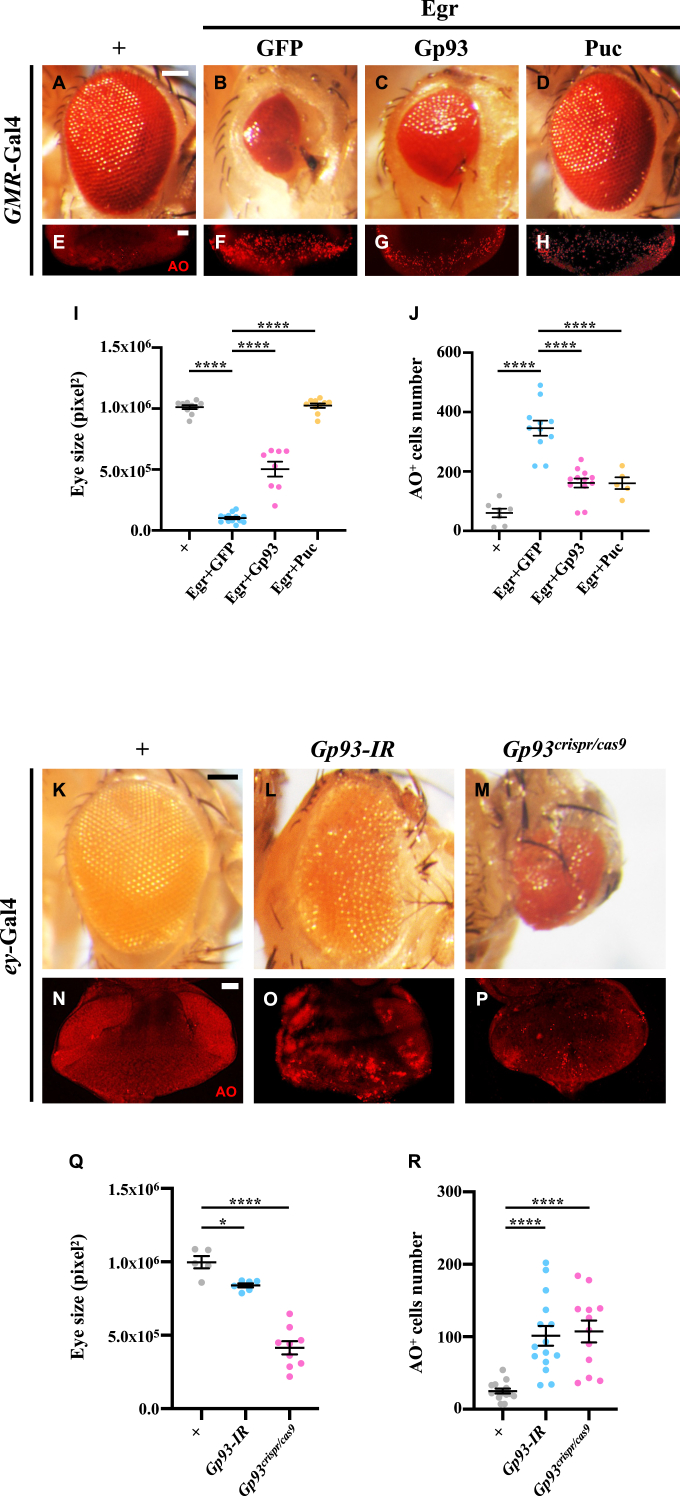
**Gp93 inhibits cell death in eye development.** Light micrographs of *Drosophila* adult eyes (A-D, K-M) and fluorescent micrographs of third instar larval eye-antennal discs (E-H, N–P) are shown. Compared with the *GMR*-Gal4 control (A, E), *GMR* > Egr*-*induced small eyes (B) and increased AO staining (F) were suppressed by the overexpression of Gp93 (C, G) or Puc (D, H). Compared with the *ey*-Gal4 control (K, N), eye specific knockdown (L, O) or knockout (M, P) of *Gp93* induced smaller eyes (K–M) and increased AO staining (N–P). Statistical analyses of eyes size for A-D (I) and K-M (Q), and AO staining for E-H (J) and N–P (R) were performed using a one-way ANOVA with post hoc Dunnett's multiple comparisons test. ∗*P* < 0.05 and ∗∗∗∗*P* < 0.0001. Scale bar: 100 μm in A-D and K-M, 20 μm in E-H and N–P.

To identify novel regulators of the JNK signaling, our laboratory has been utilizing a *Drosophila* model with a small eye phenotype, induced by the ectopic expression of the tumor necrosis factor (TNF) ortholog Egr driven by *GMR*-GAL4 (*GMR* > Egr) [24]. This approach has unveiled novel modulators of the JNK signaling [[24], [25], [26], [27], [28], [29]]. As reported previously [28], *GMR* > Egr flies displayed reduced eye size in adults and extensive cell death, as indicated by acridine orange (AO) staining, in the 3rd instar larval eye discs, when compared with the controls (Fig. 1 A, B, E, F, I and J). We found that overexpression of Gp93 significantly suppressed the small eye phenotype and AO staining induced by *GMR* > Egr (Fig. 1C–G, I and J). Overexpression of Puc, a serine/threonine protein phosphatase that dephosphorylates and inhibits JNK [15], served as a positive control (Fig. 1D–H, I and J). These findings suggest that overexpression of Gp93 inhibits ectopic Egr-triggered cell death in eye development.

To investigate the physiological role of *Gp93* in cell death, we utilized eye-specific Gal4 drivers to achieve *Gp93* knockdown via RNA interference (RNAi) and conditional knockout by CRISPR/Cas9 technology. Loss of *Gp93* resulted in increased cell death in eye discs and small eyes in adults (Fig. 1K-R, Fig. S1). The efficacy of *Gp93* knockdown or knockout was validated (Fig. S2). While overexpression of Gp93 alone did not affect eye size (Fig. S3), it rescued the small eye phenotype induced by *Gp93* knockdown (Fig. S4). To overcome the potential off-target effects of the knockdown and knockout techniques, we employed the *ey* > Flp; FRT *GMR*-Hid method [30] to generate homozygous *Gp93*^*3*^ mutant eyes in otherwise heterozygous flies. *Gp93*^*3*^ mutant eyes were significantly reduced in size, rescued by adding one copy of *Gp93*^*Rescue*^, a genomic rescue transgene for *Gp93* (Fig. S5). Collectively, these results suggest that *Gp93* is physiologically required to inhibit cell death in eye development.

### 2.2 Gp93 impedes cell death in wing development

**Fig. 2:**
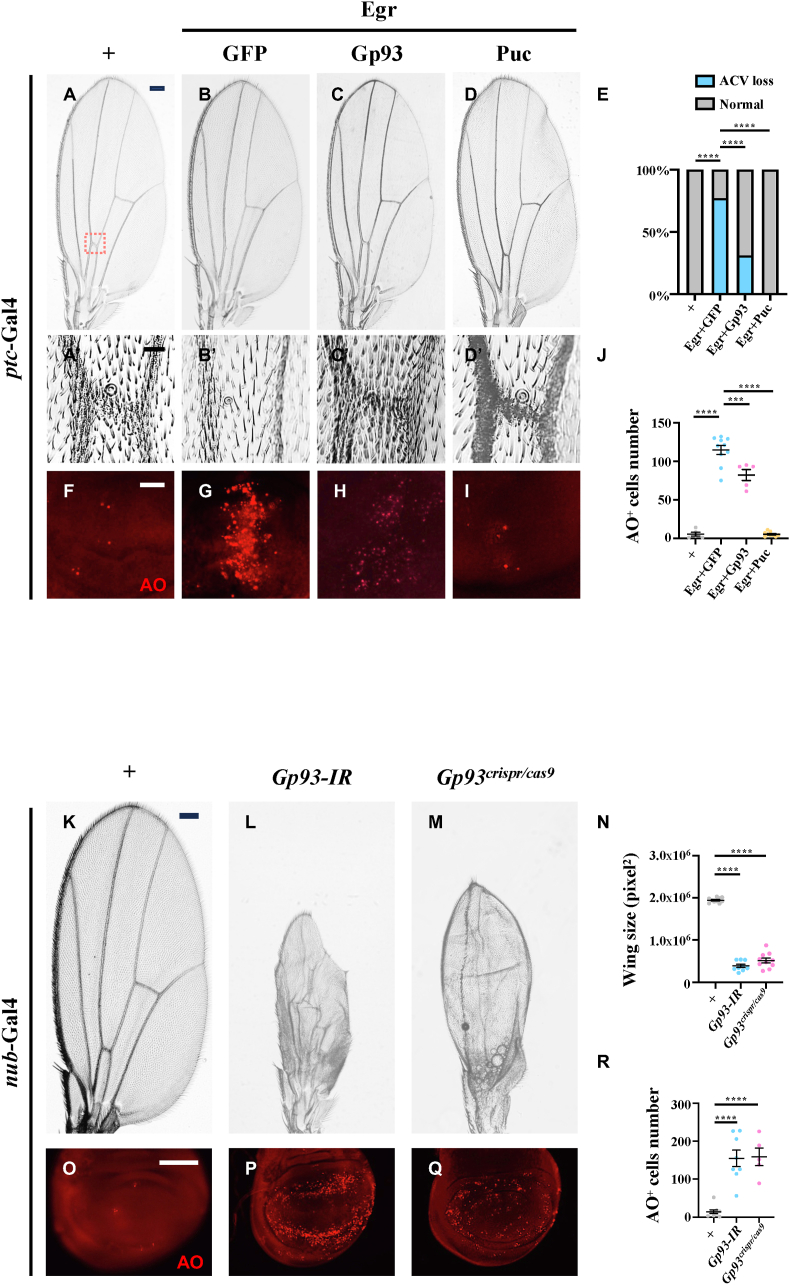
**Gp93 impedes cell death in wing development.** Light micrographs of *Drosophila* adult wings (A-D, K-M) and fluorescent micrographs of third instar larval wing discs (F–I and O-Q) are shown. Compared with the *ptc*-Gal4 control (A, A′ and F), *ptc* > Egr*-*induced ACV loss (B, B′) and increased AO staining (G) were suppressed by overexpression of Gp93 (C, C'and H) or Puc (D, D' and I). Red dashed box indicates the region of ACV. (A′-D′) Magnification of ACV region. (E) Statistical analysis of ACV loss was performed using Fisher's exact test, ∗∗∗∗*P* < 0.0001. (J) Statistical analysis of AO staining was performed using a one-way ANOVA with post hoc Dunnett's multiple comparisons test. ∗∗∗*P* < 0.001 and ∗∗∗∗*P* < 0.0001. Compared with the *nub*-Gal4 control, wing pouch specific knockdown or knockout of *Gp93* induced smaller wings (K–M) and enhanced AO staining (O–Q). Statistical analyses of wing size (N) and AO staining (R) were performed using a one-way ANOVA with post hoc Dunnett's multiple comparisons test. ∗∗∗∗*P* < 0.0001. Scale bar: 100 μm in A-D and K-M, 20 μm in A′-D′ and F–I, 50 μm in O-Q.

To explore the tissue specificity of Gp93's function, we overexpressed first Egr along the anterior/posterior (A/P) compartment boundary in larval wing discs by *ptc*-Gal4 (*ptc* > Egr). We observed the loss of the anterior cross vein (ACV) in adult wings and extensive cell death in larval wing discs when compared with controls (Fig. 2A-A′, B–B′, E-G and J). Overexpression of Gp93 significantly suppressed ectopic Egr-induced cell death and ACV loss, with Puc expression as a positive control (Fig. 2C-C′, D-D′, E and H-J). Conversely, knockdown of *Gp9*3 by *ptc*-Gal4 increased cell death along the A/P boundary in larval wing discs (Fig. S6). Due to the broad expression of *ptc*-Gal4 in other tissues that includes the central nervous system, salivary gland, fat body, midgut, and malpighian tubules, these larvae failed to reach the adult stage. Using *nub*-Gal4 to specifically deplete *Gp93* in the wing pouch, we observed a significant reduction in adult wing size (Fig. 2K-N) and increased cell death in the pouch area of larval wing discs (Fig. 2O-R). These results confirm that Gp93 prevents cell death in *Drosophila* wing development.

### 2.3 Depletion of *Gp93* induces apoptotic cell death

**Fig. 3:**
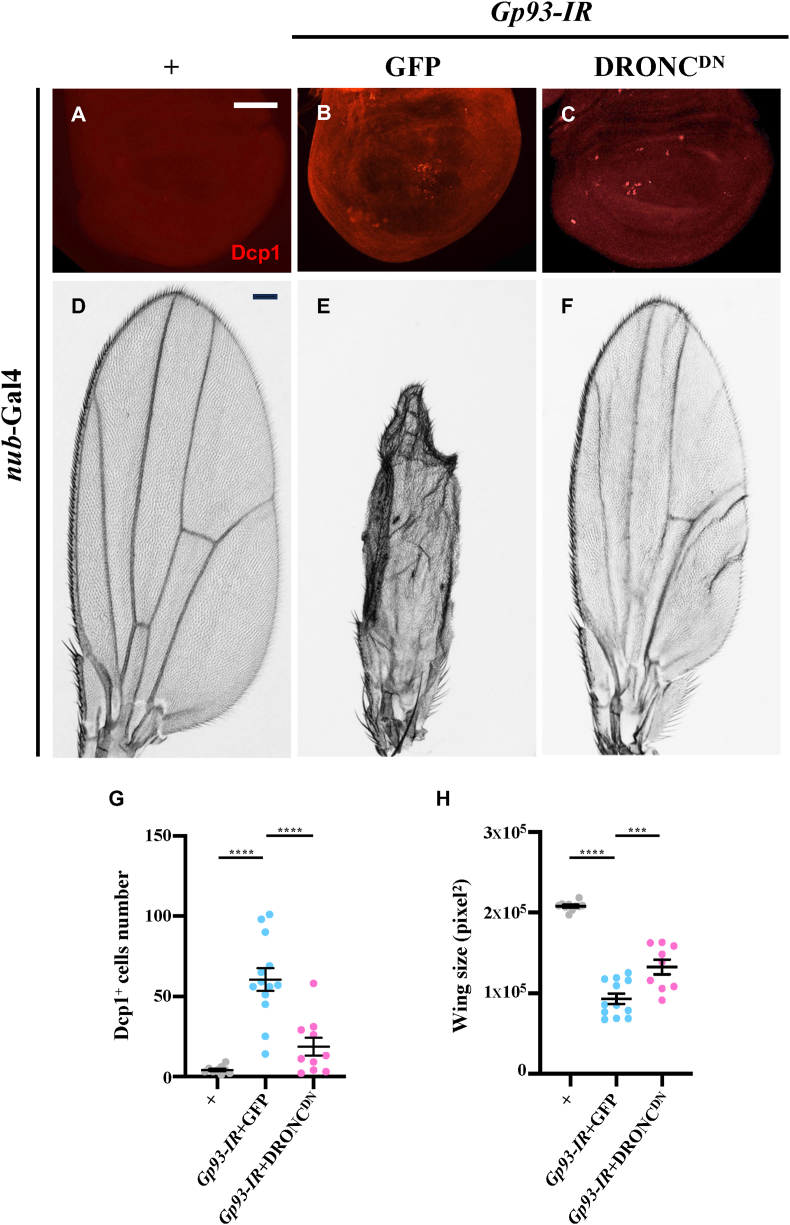
**Depletion of*****Gp93*****triggers apoptosis.** Fluorescent micrographs of *Drosophila* third instar larval wing discs (A–C) and light micrographs of *Drosophila* adult wings (D–F) are shown. Compared with the *nub*-Gal4 control, *Gp93* depletion-activated cleaved-Dcp1 staining was suppressed by expression of DRONC^DN^ (A–C). Compared with the *nub*-Gal4 control, depletion of *Gp93*-induced wing size reduction was suppressed by expression of DRONC^DN^ (D–F). Statistical analyses of ACV loss (G) and wing size (H) were performed using a one-way ANOVA with post hoc Dunnett's multiple comparisons test, ∗∗∗*P* < 0.001 and ∗∗∗∗*P* < 0.0001. Scale bar: 50 μm in A-C, 100 μm in D-F.

Recognizing apoptosis as an essential form of cell death in *Drosophila* [31], we assessed apoptosis using antibody against cleaved Dcp1 or cleaved caspase3 (C-cas3). *Gp93* depletion resulted in elevated staining for cleaved Dcp1 (Fig. 3A, B and G) and C-cas3 (Figs. S7A–C), as well as upregulated transcription of *reaper* (*rpr*) that encodes an initiator of the apoptotic pathway (Fig. S7D). More importantly, *Gp93* depletion-induced apoptosis in the larval wing pouch and wing size reduction were substantially suppressed by expressing a dominant negative form of DRONC (DRONC^DN^) (Fig. 3C, F–H). These results suggest that *Gp93* inhibits apoptosis in development.

### 2.4 *Gp93* prevents physiological JNK-mediated apoptosis

**Fig. 4:**
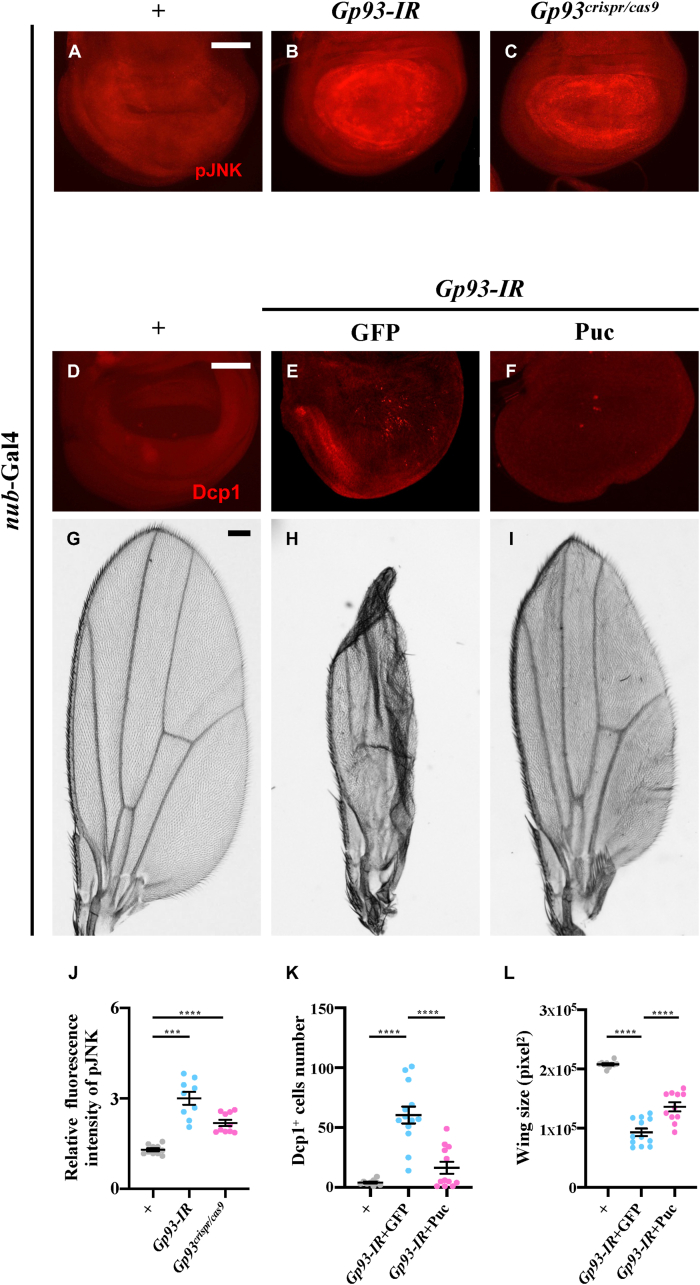
**Depletion of*****Gp93*****triggersJNK-mediated apoptosis.** Fluorescent micrographs of *Drosophila* third instar larval wing discs (A-C, D-F) and light micrographs of *Drosophila* adult wings (G–I) are shown. Compared with the *nub*-Gal4 control, *Gp93* depletion activated phosphorylation of JNK (A–C). Compared with the *nub*-Gal4 control, *Gp93* depletion-activated cleaved-Dcp1 staining and smaller wings were suppressed by Puc expression (D–I). Statistical analyses of pJNK staining (J), Dcp1 staining (K) and wing size (L) were performed using a one-way ANOVA with post hoc Dunnett's multiple comparisons test. ∗∗∗*P* < 0.001 and ∗∗∗∗*P* < 0.0001. Scale bar: 50 μm in A-F and 110 μm in G-I.

Given that Gp93 interferes with both Egr-triggered cell death and developmental apoptosis, we hypothesized that *Gp93* depletion triggers apoptosis through the activation of JNK signaling. Consistently, loss of *Gp93* resulted in elevated JNK phosphorylation (Fig. 4A–C, J and Fig. S8) and increased expression of *puc* mRNA and protein (Fig. S9), both of which are indicative of JNK signaling activation. Overexpression of Puc, a JNK inhibitor, significantly suppressed *Gp93* depletion-induced apoptosis (Fig. 4D–F, K) and wing size reduction (Fig. 4G–I, L), indicating that loss of *Gp93* triggers JNK-mediated apoptosis.

To determine whether Gp93 overexpression could impede physiological JNK-mediated apoptosis, we examined the effects of Gp93 overexpression on apoptosis induced by the loss of *scribble* (*scrib*), a protein crucial for maintaining epithelial cell polarity [32]. In the absence of *scrib*, cells undergo apoptosis triggered by the intrinsic Egr-JNK pathway [33,34]. In line with this, *ptc*-Gal4-driven *scrib* depletion resulted in ACV loss in adult wings and elevated apoptosis in larval wing discs, which were effectively suppressed by expressing Gp93 (Fig. S10). These findings propose that Gp93 inhibits physiological JNK signaling-induced apoptosis in development.

### 2.5 *Gp93* depletion induces JNK-dependent apoptosis through ROS

**Fig. 5:**
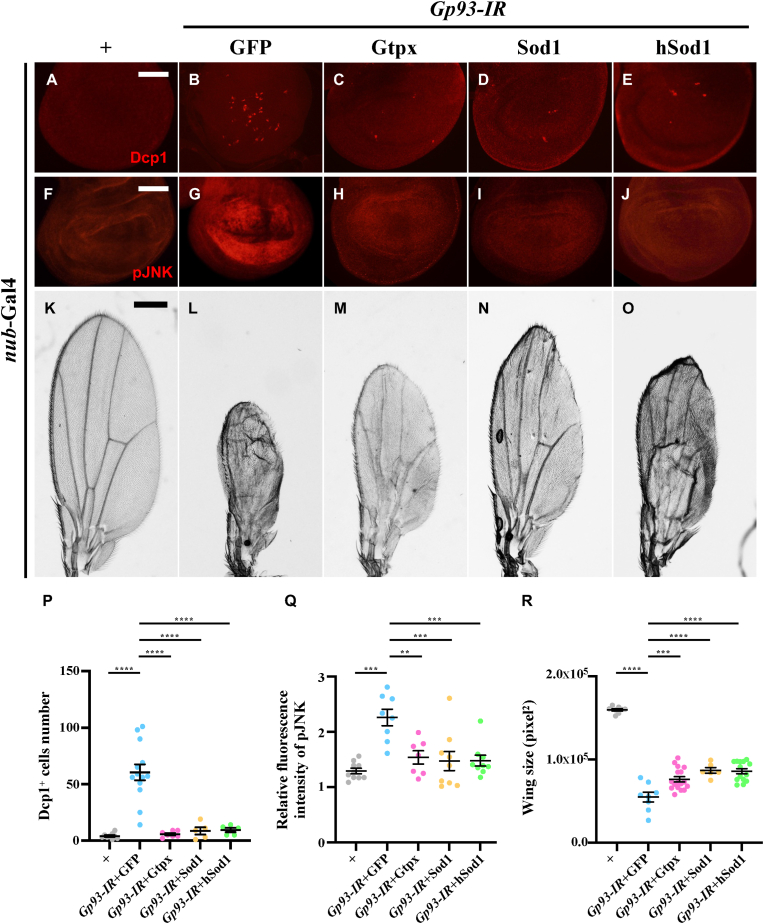
**Loss of *Gp93* triggers ROS-mediated JNK activation and apoptosis.** Compared with the *nub*-Gal4 control, *Gp93* depletion-induced Dcp1 staining and pJNK staining were suppressed by overexpression of Gtpx, Sod1 or hSod1 (A–J). Compared with the *nub*-Gal4 control, *Gp93* depletion-induced smaller wings were suppressed by overexpression of Gtpx, Sod1 or hSod1 (K–O). Statistical analyses of Dcp1 staining (P), pJNK staining (Q) and wing size (R) were performed using a one-way ANOVA with post hoc Dunnett's multiple comparisons test. ∗∗*P* < 0.01, ∗∗∗*P* < 0.001 and ∗∗∗∗*P* < 0.0001. Scale bar: 50 μm in A-J and 250μm in K–O.

Given that JNK signaling-induced apoptosis can be triggered by oxidative stress [35], we investigated whether ROS is involved in *Gp93* depletion-induced JNK-dependent apoptosis. We observed that *Gp93* knockdown in the wing pouch led to increased dihydroethidium (DHE) staining (Figs. S11A–C), indicative of elevated ROS levels [36], and a decrease in the mRNA expression of *Sod1* and *Cat* (Fig. S11D) that encode antioxidant enzymes. Furthermore, *Gp93* depletion-induced JNK activation and apoptosis in larval wing discs, as well as the smaller wing phenotype in adults, were significantly suppressed by overexpressing antioxidant proteins Gtpx, Sod1, or the human ortholog of Sod1 (hSod1) (Fig. 5A-R). These results collectively imply that the depletion of *Gp93* induces ROS-mediated JNK-dependent apoptosis.

### 2.6 Gp93 acts as an ER chaperone to prevent ROS-JNK-induced apoptosis

To determine whether Gp93's role in preventing ROS-JNK-mediated apoptosis depends on its chaperone function in the endoplasmic reticulum (ER), we overexpressed two distinct chaperones in the background of *Gp93* depletion. BiP, a member of the heat shock protein 70 (HSP70) family, is an ER specific chaperone, while heat shock protein 83 (Hsp83), a paralog of Gp93, is a cytoplasmic chaperone. We found that overexpression of BiP, but not Hsp83, rescued the reduced wing size phenotype induced by *Gp93* knockdown (Fig. S12). Consistently, *Gp93* depletion-induced ROS induction, JNK activation, and cell death were significantly alleviated by BiP overexpression (Fig. S13). These data suggest that the chaperone function of Gp93 in the ER is specifically required to prevent ROS-JNK-induced apoptosis in development.

### 2.7 *Gp93* depletion induces UPR-mediated ROS production and apoptosis

**Fig. 6:**
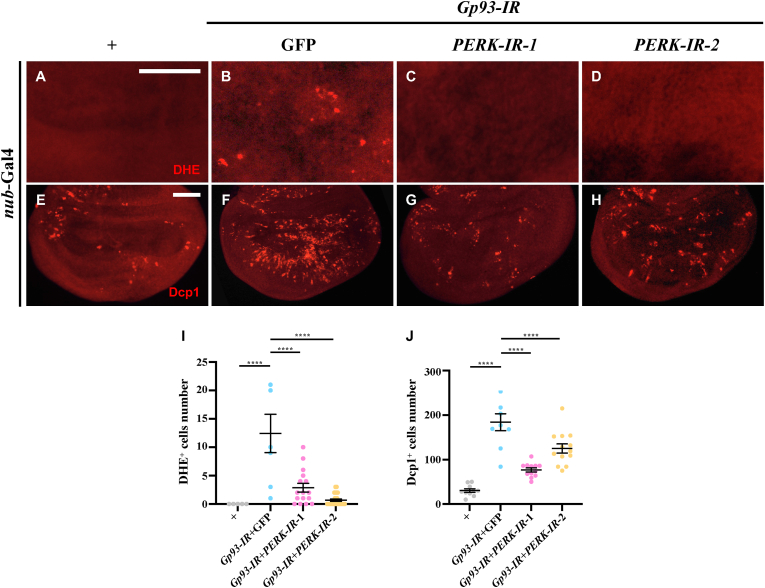
**PERK is required for*****Gp93*****depletion-induced ROS and apoptosis.** Compared with *nub*-Gal4 control (A, E), *Gp93* depletion-induced DHE staining (B) and Dcp1 staining (F) were suppressed by knockdown of *PERK* (C-D, G-H). Statistical analyses of DHE staining (I) and Dcp1 staining (J) were performed using a one-way ANOVA with post hoc Dunnett's multiple comparisons test. ∗∗∗∗*P* < 0.0001. Scale bar: 50 μm in A-H.

As Gp93 functions as an ER chaperone that aids in the proper folding of other proteins, we postulated that reduced Gp93 level might trigger the unfolded protein response (UPR). Indeed, we found that *Gp93* knockdown enhanced the phosphorylation of eIF2α (Figs. S14A and B), a marker of UPR activation mediated by pancreatic eIF-2 α kinase (PERK). PERK is known to induce ROS generation [37], and consistently, we found that overexpression of PERK increased DHE staining (Figs. S14C–E). Importantly, the knockdown of *PERK* attenuated *Gp93* depletion-induced ROS and apoptosis (Fig. 6A–J), suggesting that loss of *Gp93* promotes UPR-mediated ROS generation and apoptosis.

### 2.8 Gp93 protects neuronal cells from ROS-mediated JNK-dependent neurodegeneration

**Fig. 7:**
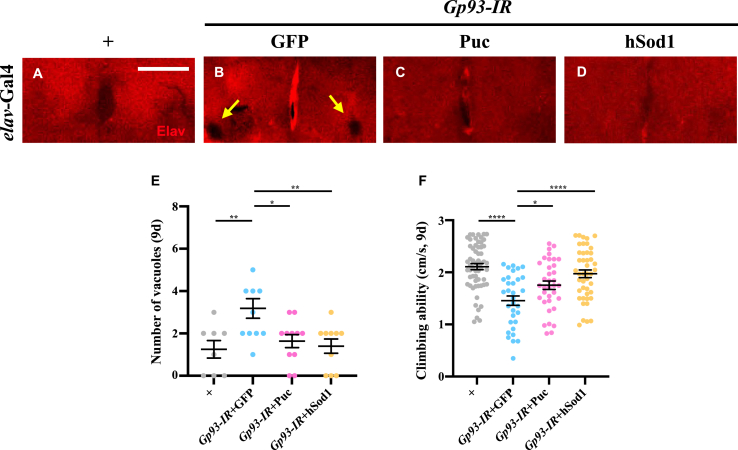
**Depletion of*****Gp93*****triggers ROS-JNK-mediated neurodegeneration.** Fluorescent micrographs of 9-day-old *Drosophila* brains are shown (A–D). Compared with the *elav*-Gal4 control (A), depletion of *Gp93* resulted in the formation of brain vacuoles, indicated by arrows (B). These vacuoles were suppressed by the overexpression of Puc (C) or hSod1 (D). Statistical analysis of vacuole numbers (E) was performed using a one-way ANOVA with post hoc Dunnett's multiple comparisons test. ∗*P* < 0.05 and ∗∗*P* < 0.01. The average climbing speed of 9-day-old flies was measured by recording the distance climbed from the bottom of a tube within 5 s (F). Compared with the control, neuron-specific depletion of *Gp93* caused a significant reduction in climbing speed, which was rescued by overexpression of Puc or hSod1. Statistical analysis of climbing ability was performed using a one-way ANOVA with post hoc Dunnett's multiple comparisons test. ∗*P* < 0.05 and ∗∗∗∗*P* < 0.0001. Scale bar: 50 μm in A-D.

Excessive ROS production is a well-established cause of disrupted neuronal function [38]. To explore the role of *Gp93* in averting ROS-mediated neuronal cell death and preserving neuronal function, we depleted *Gp93* specifically in neurons. This intervention led to reduced brain size and vacuolization (Figs. S15A–D), hallmarks of neuronal cell death and neurodegeneration [[39], [40], [41]]. Evaluation of the climbing ability in adult flies [42] revealed a discernible climbing defect in the *Gp93*-depleted group compared with the control group (Fig. S15 E). Moreover, both vacuolization and the climbing defect were mitigated by concurrent expression of Puc or hSod1 (Fig. 7A–F), suggesting that *Gp93* depletion triggers ROS-JNK-mediated neurodegeneration.

**Fig. 8:**
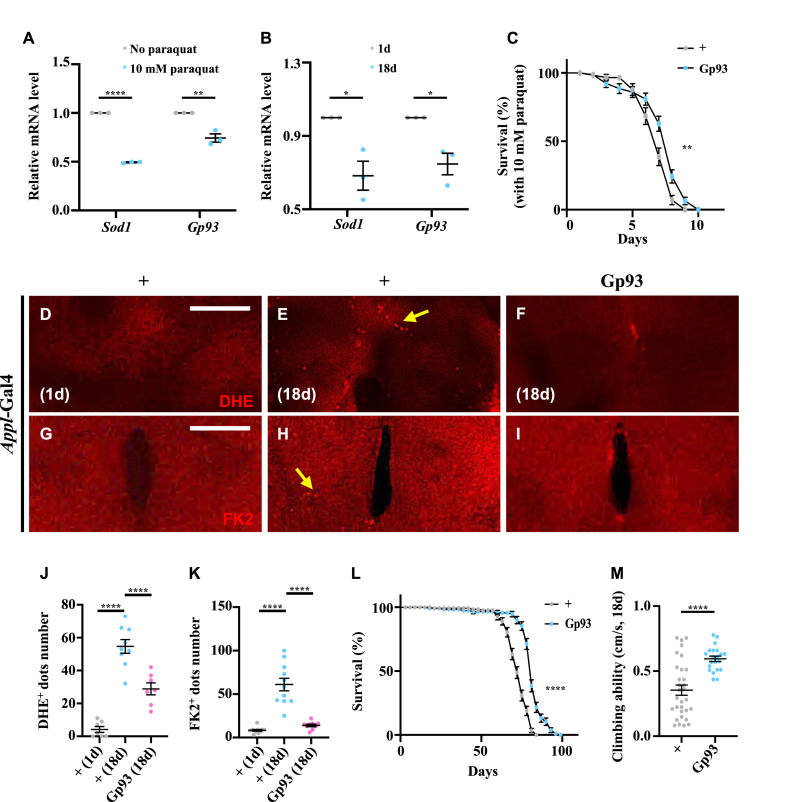
**Gp93 overexpression alleviates paraquat or aging-induced ROS-mediated neurodegeneration.** A 10 mM paraquat treatment significantly reduced the mRNA levels of *Sod1* and *Gp93* in fly brains compared with controls (A). Brains from 18-day-old flies exhibited significantly lower *Sod1* and *Gp9*3 mRNA levels compared with 1-day-old controls (B). Statistical analyses were conducted using an unpaired *t*-test, ∗*P* < 0.05, ∗∗*P* < 0.01 and ∗∗∗∗*P* < 0.0001. The reduced lifespan caused by paraquat treatment was partially rescued by neuron-specific overexpression of Gp93 driven by *Appl*-Gal4 (C). Statistical analysis was conducted with the Log-rank (Mantel-Cox) test, ∗∗*P* < 0.01. Compared with 1-day-old brains (D, G), 18-day-old brains displayed elevated DHE staining (E, arrow) and FK2 staining (H, arrow), which were suppressed by overexpression of Gp93 (F, I). Statistical analysis for DHE (J) and FK2 (K) staining were performed using a one-way ANOVA with post hoc Dunnett's multiple comparisons test, ∗∗∗∗*P* < 0.0001. Neuron-specific overexpression of Gp93 driven by *Appl*-Gal4 extended fly lifespan (L). Statistical analysis was conducted with the Log-rank (Mantel-Cox) test, ∗∗∗∗*P* < 0.0001. Compared with controls, 18-day-old flies with neuron-specific overexpression of Gp93 displayed enhanced climbing speed (M). Statistical analysis was conducted with an unpaired *t*-test, ∗∗∗∗*P* < 0.0001. Scale bar: 25μm in D-I.

ROS-mediated neurodegeneration has also been associated with paraquat treatment, a known inducer of oxidative stress, and aging [43,44]. To further validate the protective effect of Gp93 against ROS-induced neurodegeneration, we tested whether Gp93 overexpression could alleviate the neurodegeneration symptoms caused by paraquat or aging. Consistent with its physiological role in preventing ROS-mediated neurodegeneration, we observed that the mRNA level of *Gp93*, as that of *Sod1*, was significantly decreased upon paraquat treatment (Fig. 8A) or in aged brains (18-day-old vs 1-day-old, Fig. 8B), implying that *Gp93* expression is negatively regulated by ROS. Intriguingly, neuron-specific overexpression of Gp93, driven by *Appl*-Gal4 or *elav*-Gal4, prolonged survival in flies exposed to paraquat (Fig. 8C, Fig. S16A), suggesting that ectopic Gp93 antagonizes paraquat-induced oxidative stress.

Compared with 1-day-old flies, the brains of 18-day-old flies exhibits increased ROS level revealed by DHE staining, which was mitigated by Gp93 overexpression (Fig. 8D–F and J). Moreover, we observed impaired protein homeostasis in aging brains, as indicated by increased FK2 staining for ubiquitinated proteins, which was suppressed by Gp93 overexpression (Fig. 8G–I and K). Additionally, Gp93 overexpression significantly extended the lifespan of flies (Fig. 8L, Fig. S16B) and improved the climbing ability (Fig. 8M, Fig. S16C). However, it did not affect mitochondrial membrane potential as assessed by the JC-1 mitochondrial membrane potential assay, a parameter closely associated with mitochondrial function [45,46] (Fig. S16D). These results advocate for the critical role of Gp93 in sustaining normal neuro function by preventing aging-associated ROS-mediated neurodegeneration.

### 2.9 HSP90B1 preserves Gp93's function in preventing ROS-JNK-mediated apoptosis in *Drosophila* development

**Fig. 9:**
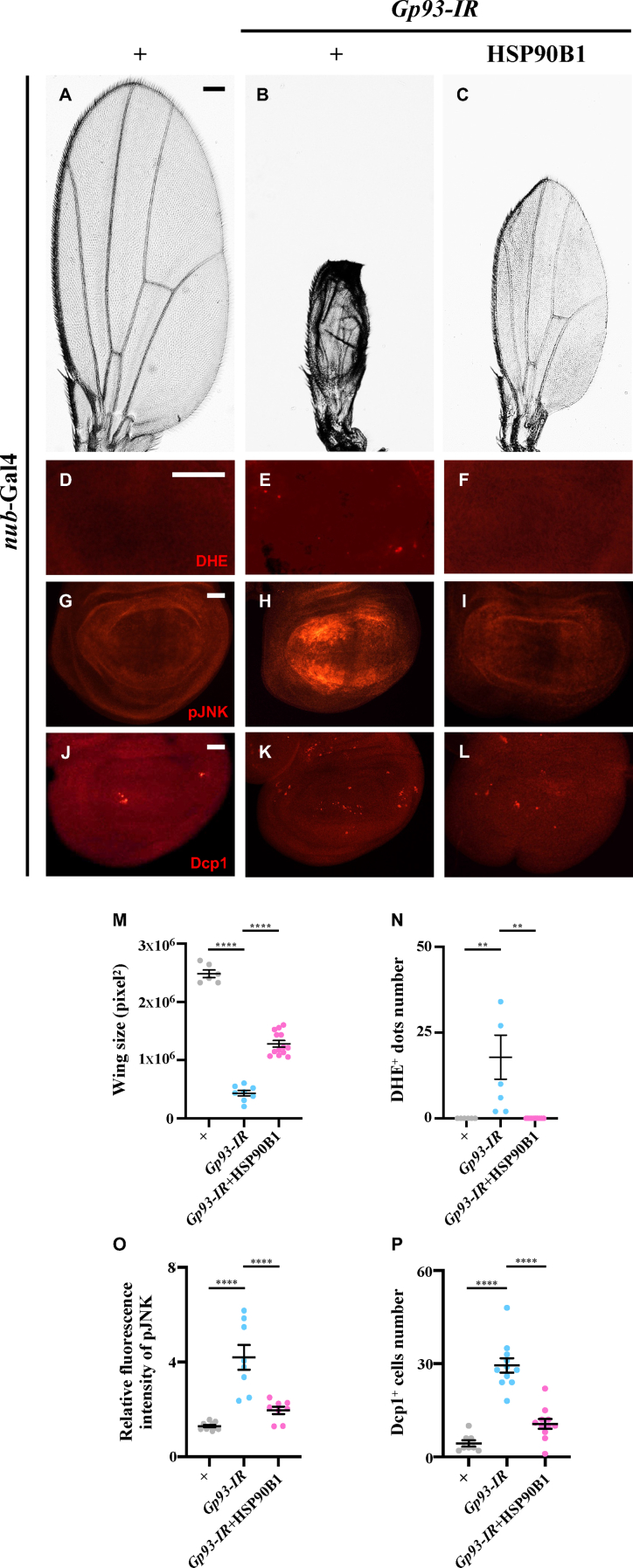
**HSP90B1 retains Gp93's function in preventing ROS-JNK signaling-mediated apoptosis.** Compared with the *nub*-Gal4 control (A, D, G and J), *Gp93* depletion-induced wing size reduction (B) and elevated staining of DHE (E), pJNK (H) and cleaved Dcp1 (K) were significantly suppressed by HSP90B1 overexpression (C, F, I and L). Statistical analysis of wing size (M), DHE staining (N), pJNK staining (O) and Dcp1 staining (P) were performed using a one-way ANOVA with post hoc Dunnett's multiple comparisons test. ∗∗*P* < 0.01 and ∗∗∗∗*P* < 0.0001. Scale bar: 100 μm in A-C, 50 μm in D-F and 25 μm in G-L.

HSP90B1, the human ortholog of Gp93, has emerged as a significant contributor to cancer development [21]. To explore whether HSP90B1 preserves Gp93's role in impeding ROS-JNK-mediated apoptosis, we introduced HSP90B1 into *Drosophila*. We employed *nub*-Gal4 to knock down *Gp93* in the developing wing pouch, and observed that expression of HSP90B1 effectively rescued *Gp93* depletion-induced small wing phenotype in adult flies, as well as ROS induction, JNK activation, and apoptosis in the larval wing discs (Fig. 9A-P, Fig. S17). These findings suggest that HSP90B1 retains Gp93's capacity to inhibit ROS-JNK-mediated apoptosis, thereby emphasizing the evolutionary conservation of this critical function.

### 2.10 *HSP90B1* depletion induces ROS, JNK activation and apoptosis in human cells

**Fig. 10:**
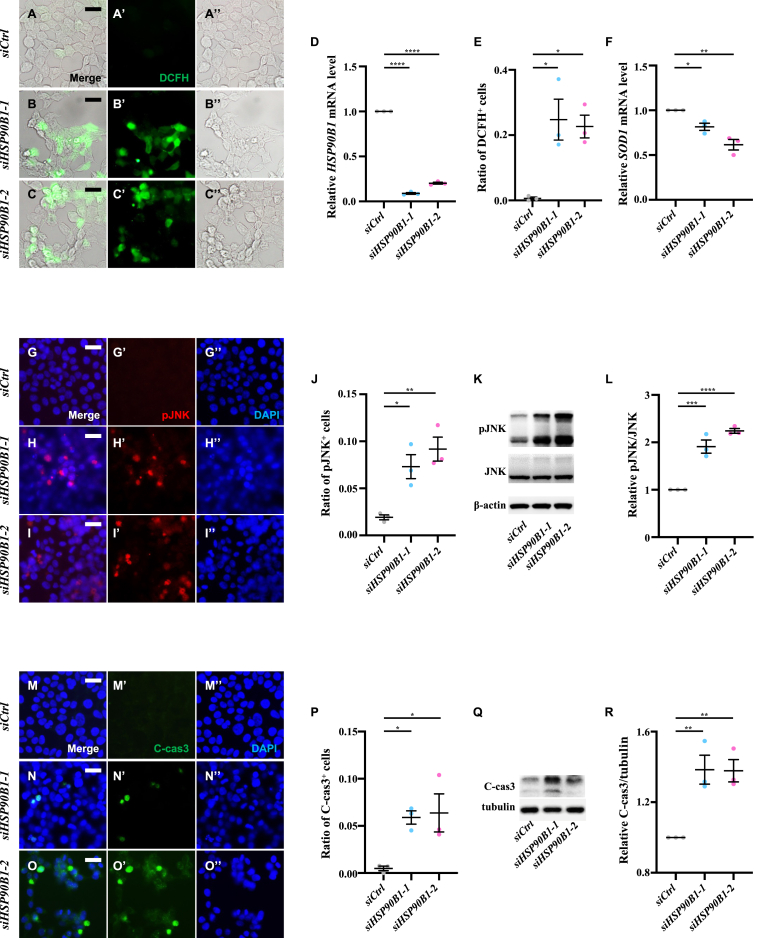
**Depletion of*****HSP90B1*****induces ROS generation, JNK activation and apoptosis in human cells.** Compared with controls (A-A″), knockdown of *HSP90B1* in human 293T cells increased DCFH staining, indicating elevated ROS levels (B–B″ and C–C″). (D) The knockdown efficacy of two RNAi targeting *HSP90B1* was validated via RT-qPCR. Statistical analysis was performed using a one-way ANOVA with post hoc Dunnett's multiple comparisons test, ∗∗∗∗*P* < 0.0001. (E) Statistical analysis of DCFH staining was performed using a one-way ANOVA with post hoc Dunnett's multiple comparisons test, ∗*P* < 0.05. (F) Knockdown of *HSP90B1* reduced *SOD1* mRNA levels, with statistical analysis performed using a one-way ANOVA with post hoc Dunnett's multiple comparisons test, ∗*P* < 0.05 and ∗∗*P* < 0.01. Compared with controls (G-G″), knockdown of *HSP90B1* induced pJNK activation in 293T cells (H–H″ and I–I″). Immunoblot assays confirmed that *HSP90B1* knockdown increased pJNK levels without affecting total JNK protein (K). Statistical analysis of pJNK-positive cells (J) and pJNK protein level (L) were performed using a one-way ANOVA with post hoc Dunnett's multiple comparisons test, ∗*P* < 0.05, ∗∗*P* < 0.01, ∗∗∗*P* < 0.001 and ∗∗∗∗*P* < 0.0001. Compared with controls (M-M″), knockdown of *HSP90B1* induced C-cas3 staining, indicating apoptosis (N–N″ and O–O″). (Q) Immunoblot assays showed increased production of C-cas3 following *HSP90B1* knockdown. Statistical analysis of C-cas3 positive cells (P) and C-cas3 level (R) were performed using a one-way ANOVA with post hoc Dunnett's multiple comparisons test. ∗*P* < 0.05 and ∗∗*P* < 0.01. Scale bar: 50 μm A-C″, G-I″ and M-O″.

To ascertain whether HSP90B1 plays a conserved role in ROS production, JNK activation, and apoptosis in mammalian cells, we silenced *HSP90B1* expression in human 293T cells. Compared with controls, *HSP90B1* knockdown led to elevated ROS levels, as indicated by dichlorodihydrofluorescein diacetate (DCFH-DA) staining, and reduced *SOD1* mRNA expression (Fig. 10A–F). Moreover, *HSP90B1* knockdown enhanced pJNK staining (Fig. 10G–J) and elevated pJNK protein levels (Fig. 10K and L). Finally, *HSP90B1* knockdown induced apoptosis labeled with Annexin V-FITC (Fig. S18) and C-cas3 staining (Fig. 10M-P), as well as increased levels of C-cas3 protein (Fig. 10Q and R). These results suggest that HSP90B1 is required to prevent ROS accumulation, JNK activation, and apoptosis in human cells.

## 3 Discussion

Heat shock proteins (HSPs), a highly conserved family of stress response proteins, are critical for cellular homeostasis. They assist in proper proteins folding, prevent protein aggregation, and promote the degradation of misfolded proteins – all essential functions for cells navigating stressful conditions [47]. For example, during oxidative stress, HSP70 not only acts as a chaperone for misfolded proteins associated with apoptotic cell death pathway, thereby inhibiting cell death, but also regulates the expression of other heat shock proteins in eukaryotic cells [48]. Similarly, HSP90B1, the mammalian ortholog of *Drosophila* Gp93, contributes to antioxidant function in oxidative stress scenarios [22,23]. A key question arises: what happens when heat shock proteins are disturbed in the absence of stress, considering they are typically expressed at lower levels under normal condition? To date, the role of Gp93/HSP90B1 in such a context remains unexplored. Our findings indicate that the loss of *Gp93* induces oxidative stress and JNK-dependent apoptotic cell death, highlighting the crucial role of heat shock proteins in maintaining tissue homeostasis even under non-stressful conditions.

Although our study identified Gp93's protective role against apoptosis, we did not delve into other forms of cell death. Existing research suggests that oxidative stress can also induce necrotic cell death [49], and we observed phenotypes associated with necrotic cell death in *Gp93*-depleted flies (data not shown). We speculate that Gp93 may also confer protection against necrotic cell death, and we plan to investigate this further. Unraveling this novel mechanistic link between Gp93 and necrotic cell death will deepen our understanding of the diverse cell survival mechanisms orchestrated by Gp93, shedding light on its multifaceted role in cellular homeostasis.

Excessive ROS production is associated with a range of neurodegenerative disorders, such as Alzheimer's disease [50], Parkinson's disease [51], Huntington's disease [52], and amyotrophic lateral sclerosis (ALS) [53]. However, ROS typically emerge as a secondary event following the onset of pathology triggered by other factors [54,55]. Therefore, pinpointing the early contributors to ROS generation in neurodegeneration is of paramount importance. Several factors contribute to ROS production in the aging brain [56], including the depletion of redox defense systems [57,58], mitochondrial dysfunction [59], lipid peroxidation [60,61], and the accumulation of misfolded proteins [62]. The latter is a common feature in many neurodegenerative diseases. HSPs play a crucial role in the folding of newly synthesized proteins, the refolding of metastable proteins, and the degradation of misfolded proteins [63]. While most HSPs are localized in the cytosol and mitochondria, a subset is found in the ER [63]. For example, HSP5A, an ER chaperone from the HSP70 family, has been shown to mitigate PD-like neurodegeneration [64] and reduce the toxicity of ALS-related TDP-43 [65]. In contrast, HSP90B1, another ER chaperone, has not been clearly linked to neurodegenerative processes.

Our results, however, demonstrate that the depletion of *Gp93* in the *Drosophila* nervous system results in ROS-mediated JNK-dependent neuronal cell death, which impairs locomotor activity (Fig. 7). Conversely, overexpression of Gp93 enhances resistance to oxidative stress, boosts climbing ability, and extends lifespan (Fig. 8). These results underscore the importance of ER chaperones in maintaining neuronal function and suggest that ER chaperones may play a pivotal role as early factors in the pathogenesis of neurodegeneration, preceding ROS production. Future studies could explore the potential of *Gp93* knockdown flies as a model to identify early risk factors involved in ROS generation during neurodegenerative processes.

Oxidative stress is a major factor in the development of various neurodegenerative diseases, making antioxidants a promising therapeutic option [66]. However, antioxidants have shown limited effectiveness in clinical application [67]. This limitation may be due to the complex pathological mechanisms underlying these diseases, which may require a more multifaceted therapeutic approach. Additionally, in the late stages of neurodegeneration, once significant neuronal damage has occurred, antioxidants alone may not be sufficient to reverse the damage.

Our study reveals that the reduction of *HSP90B1* in human 293T cells leads to increased ROS production, JNK activation, and apoptosis, while transgenic expression of HSP90B1 inhibits these processes in *Drosophila*. Additionally, we observed a decrease in *Gp9*3 mRNA levels in aging fly brains, suggesting that human HSP90B1 may serve as a biomarker for the early stages of neurodegenerative diseases. Combining HSP90B1 administration with antioxidants may enhance therapeutic efficacy, particularly in the early stages of neurodegeneration. Future research should explore whether the loss of HSP90B1 contributes to ROS-induced neurodegeneration, whether overexpression of HSP90B1 can prevent ROS-mediated damage and mitigate neurodegeneration in mammals, and whether HSP90B1 exerts differential effects across various neurodegenerative conditions.

## 4 Methods

### 4.1 *Drosophila* husbandry and genetics

Flies were cultured on a standardized yeast/molasses-based diet consisting of 10 L water, 100 g agar, 180 g dry yeast, 250 g white sugar, 800 g corn flour, 100 g soy flour, 650 g molasses, 70 ml methyl 4-hydroxybenzoate solution (containing 2 g methyl 4-hydroxybenzoate and 7 ml ethanol), and 115 ml mixed acid solution (108.1 ml water, 83.6 ml propionic acid and 8.3 ml phosphoric acid).

The majority of flies were reared at 25°C with 60%–70% humidity under a 12 h light/dark cycle. Flies with *tub*-Gal80^ts^ at 29°C were used to negate the inhibitory effect of Gal80 on Gal4 (Fig. 2A–J, Figs. S2A, S3D, S4E, S7D, S9D, S11D, S17A). To enhance the intensity of DHE staining, the animals were kept at 29°C (Figs. S11A–B and S14C-D). To investigate the roles of PERK or BiP in the phenotypes induced by *Gp93* knockdown, animals were maintained at 29°C, as the *nub* > *Gp93-IR* line exhibited a more consistent and moderately severe pathological phenotype (Fig. 6, Figs. S12 and S13).

The fly strains used in this study include: *w*^*1118*^, *GMR*-Gal4, *UAS*-GFP, *UAS*-Puc, *UAS*-Egr, *UAS*-LacZ, *ptc*-Gal4, *UAS*-DRONC^DN^, *elav*-Gal4, *UAS*-*scrib-RNAi*, *nub-*Gal4, OR were previously described [15,24,42,68,69,70]. *UAS*-*scrib-RNAi* (V27424)*, UAS*-Gtpx (BL28479); *ey-*Gal4 (BL8220); *UAS*-Sod1 (BL24750); *UAS-*Gp93 (Kyoto206596), *UAS-Gp93-IR* (NIG5520R-3), *UAS-Gp93-IR* (NIG5520R-1), *UAS-Gp9*3-gRNA (BL83823), *UAS*-Cas9.P2 (BL67080), *UAS*-Hsp83 (BL58469), *UAS*-BiP (BL5843), *UAS*-*PERK-IR* (V110278), *UAS*-*PERK-IR* (V16427), *UAS*-Gp93 (BL16392) and *y w*; *ey*-Gal4 *UAS*-Flp; FRT*8*2 GMR-Hid CL3R/TM2 (BL5253) were obtained from indicated stock centers. Additionally, *Gp93*^*3*^and Gp93^Rescue^ were gifts from Dr. Jason C. Maynard [19], *UAS*-hSod1 was a gift from Dr. Hansong Deng, and *y w*; *ey*-Flp *act*-Gal4 *UAS*-GFP; FRT*82B tub*-Gal80 was a gift from Dr. Tian Xu. Transgenic flies expressing human HSP90B1 were generated using a PCR-amplified fragment subcloned into the pUAST vector. Puc-HA was constructed by inserting a 3 × HA tag at the C-terminal of Puc using CRISPR/Cas9 technology. All genotypes of the flies utilized in the study have been comprehensively enumerated (Table S1).

### 4.2 Cell culture and transfection

293T cells were cultivated in high-glucose Dulbecco's Modified Eagle Medium supplemented with 10% fetal bovine serum (FCS; Gibco), 100 U/ml penicillin, and 100 μg/ml streptomycin (Gibco), and maintained at 37°C in 5% CO2. All siRNAs were acquired from Hua Gene Biotechnology (Shanghai, China). Transfections were carried out using Lipofectamine RNAi MAX reagent at a concentration of 2.3 μl/ml in the culture medium, following the manufacturer's instructions (Thermo Fisher Scientific). The siRNAs used are as follow:

siCtrl (sense strand: UUCUCCGAACGUGUCACGUTT, Antisense strand: ACGUGACACGUUCGGAGAATT);

siHSP90B1-1 (sense strand: GGAAGAUGAAGAUGAUAAAtt, Antisense strand: UUUAUCAUCUUCAUCUUCCtt);

siHSP90B1-2 (sense strand: GAAGUUACCUUCAAAUCAAtt, Antisense strand: UUGAUUUGAAGGUAACUUCtt).

### 4.3 DHE staining

DHE (Sigma, 37291) staining was employed to assess ROS levels. For wing imaginal discs: freshly dissected tissues were incubated in a 10 μM DHE solution for 30 min in the dark at room temperature. After washing three times in PBS (10 min each), tissues were fixed in 4% formaldehyde for 5 min. Following another three washes in PBS (5 min each), wing discs were isolated on a glass slide under a dissecting microscope. For adult brains: Brains were dissected in Schneider's medium, incubated in 30 μM DHE solution for 30 min in the dark at room temperature. After washing twice in Schneider's medium (10 min each), brains were fixed in a freshly prepared 4% formaldehyde solution for 7 min. After three washes with PBS (5 min each), brains were placed on a glass slide under a dissecting microscope.

### 4.4 Immunostaining

**Imaginal discs**: Tissues were fixed with 4% paraformaldehyde for 15 min, then washed three times with 0.3% PBST (10 min each). After incubation with the indicated primary antibodies for 12–15 h at 4°C, tissues were washed and incubated with secondary antibody for 2 h at room temperature. Finally, tissues were washed three times in 0.3% PBST (10 min each).

**Brains**: Heads, devoid of mouths, were fixed with 4% paraformaldehyde for 1 h. Subsequently, the brains were carefully excised from the fixed heads, washed tree times in 0.3% PBST (10 min each), and incubated with primary antibodies for 12–15 h at 4°C. After primary antibody incubation, brains were washed and incubated with secondary antibodies for 2 h at room temperature. Finally, brains were washed three times in 0.3% PBST (10 min each).

**293T cells**: Cells were fixed with 4% paraformaldehyde for 20 min, permeabilized with 0.2% Triton X-100 for 1 h, and blocked with 2% BSA for 1 h. Cells were then incubated with primary antibodies for 12 h at 4 °C. After washing, cells were incubated with fluorescence dye-conjugated secondary antibodies and DAPI according to standard protocols.

All antibodies for immunostaining used are as follow: primary antibodies included Rabbit anti-phospho-JNK (Calbiochem, 559309), Rabbit anti-cleaved Caspase3 (Cell Signaling Technology, 9661), Rabbit anti-cleaved Dcp-1 (Cell Signaling Technology, 9578S), Rabbit anti-peIF2 α (Cell Signaling Technology, 3398T), Mouse anti-FK2 (Merck, 04–263), Rat anti-Elav (DSHB, 7E8A10), Rabbit anti-HA tag (Cell Signaling Technology, 3724S), Mouse anti-phospho-SAPK/JNK (Cell Signaling Technology, 9255S), Rabbit anti-cleaved caspase3 (Cell Signaling Technology, 9664S). Secondary antibodies employed were Donkey anti-Rat-Cy3 (Jackson Immuno research, 104086), Goat anti-Mouse-Cy3 (Life technologies, A11032), Goat anti-Rabbit-Cy3 (Life technologys, A11037).

### 4.5 AO staining

Wing discs were carefully dissected from third-instar larvae in 0.1% PBST and subsequently immersed in a solution containing 1 × 10^−5^ M AO for 5 min at room temperature.

### 4.6 DCFH-DA staining

DCFH-DA (Beyotime, S0033) staining was utilized to evaluate the levels of ROS in 293T cells. Cells were washed once with PBS and incubated with 10 μM DCFH-DA for 30 min at 37°C. Subsequently, cells were thoroughly rinsed three times with serum-free cell culture medium.

### 4.7 Annexin V conjugated to fluorescein isothiocyanate (annexin V-FITC) staining

The Annexin V-FITC Apoptosis Detection Kit (Beyotime, C1062) was utilized to assess apoptosis according to the manufacturer's protocol. In summary, cells were washed once with PBS and resuspended in Annexin V binding buffer. Next, 5 μL of Annexin V- FITC was introduced to the cell suspension, which was then incubated in the dark at room temperature for 10 min. It is imperative to conduct cell detection within 1 h of staining.

### 4.8 Morphology and microscopy

**Adult eyes**: Three-day-old adult flies were collected and briefly frozen for 5 min. Eye morphology was visualized using an Olympus SZX16 light microscope with an 8 × objective at a resolution of 4800✕3600 pixels.

**Adult wings**: Three-day-old adult flies were collected, frozen for 5 min, and their wings were meticulously dissected in a 1:1 mixture of ethyl alcohol and glycerin under a dissecting microscope. Slides were prepared and visualized using an Olympus DP74 light microscope with a 4 × or 20 × objective at a resolution of 5760 × 3600 pixels.

**Imaginal discs**: Approximately 5–6 days following the mating of the parent flies, wandering third instar larvae were collected as they migrate from the food source towards the tube wall. Under a dissecting microscope, eye-antennal and wing imaginal discs were carefully excised in PBS solution. Imaginal discs were visualized using an Olympus DP74 fluorescence microscope with a 20 × objective at a resolution of 5760 × 3600 pixels.

**Adult brains**: Adult brains were imaged using an Olympus FV3000 confocal microscope with a 10 × objective at a resolution of 1024 × 1024 pixels. Z-stack images were acquired at 2 μm intervals to capture the full depth of the sample.

**293T cells**: Cells were imaged using an Olympus DP74 light or fluorescence microscope with a 20 × objective at a resolution of 5760 × 3600 pixels.

### 4.9 Fluorescence excitation and emission

DAPI: Ex/Em = 340–364/454∼488 nm.

Cy3: Ex/Em = 515–545/560∼590 nm.

DHE: Ex/Em = 535/610 nm.

Annexin V-FITC: Ex/Em = 490–495/515∼530 nm.

DCFH: Ex/Em = 488/525 nm.

### 4.10 Image analysis

All images of eyes, wings, eye-antennal imaginal discs, wing imaginal discs, and brains were standardized using Adobe Photoshop 2022. The detailed methods for image analysis are described below:

**Cropping picture**: File → Open → Rectangular Marquee tool → Crop tool.

**Measuring tissue size**: (1) Open the image: File → Open; (2) Select the region of interest: Rectangular Marquee Tool → Quick Selection Tool → Record Measurements → Export the selected measurements. Data were analyzed and plotted as mean ± SEM using GraphPad Prism 9.0.

**Measuring fluorescence intensity**: (1) Open the image: File → Open; (2) Select the region of interest: Quick Selection Tool → Record Measurements → Export selected measurements; (3) Select a background region: Rectangular Marquee Tool → Record Measurements → Export selected measurements. Relative intensity = mean gray value of wing pouch area/mean gray value of background region. Values were analyzed and plotted as mean ± SEM using GraphPad Prism 9.0.

**Counting cells or dots**: File → Open → Count tool → Record Measurements → Export selected measurements. Values were analyzed and plotted as mean ± SEM using GraphPad Prism 9.0.

### 4.11 Climbing ability assay

To assess the climbing ability, newly eclosed flies (within 24 h) were reared at 25°C, 60%–70% humidity, and subjected to a 12 h light/dark cycle. For each genotype, 50–60 flies were used, with 20–25 flies per tube. Flies were transferred to fresh vials every three days to ensure optimal living conditions. On days 9 and 18, flies were placed in 14 cm-long transparent plastic tubes, which were secured to a rack for the climbing assay. The flies were vibrated to the bottom of the tube by a machine and strived to climb upward. The process was recorded on video, and a 5 s segment showing the ascent was extracted for analysis. The climbing height within this segment was measured using Adobe Photoshop 2022, and the average climbing speed was calculated.

### 4.12 Longevity assay

For each genotype, 50–60 flies were tested in groups of 20–25 per tube. The flies were reared at 25°C, 60% humidity, and subjected to a 12 h light/dark cycle. Every three days, flies were transferred to fresh vials, and their mortality was recorded until all flies had died.

### 4.13 Paraquat treatment

3-day-old flies were gathered, and 15–20 individuals were placed in each vial. After a 6 h starvation period, the flies were transferred to vials with a medium composed of 10 mM paraquat (Macklin, M919425), 4% sucrose, and 1% agar. For RT-qPCR, flies were exposed to paraquat for a duration of 12 h. Later, their heads were separated from the body under liquid nitrogen for RNA extraction. For survival analysis, flies were continuously exposed to paraquat until mortality was observed in the entire cohort.

### 4.14 JC-1 staining

Five freshly dissected brains were collected in Schneider's medium per group. Subsequently, 50 μL of a 1 mg/ml papain solution (Worthington, LK003176) was added to each tube, and the brains were incubated in a 37°C water bath for a period of 30–40 min. Following the incubation, the JC-1 staining was carried out using the JC-1 Mitochondrial Membrane Potential Assay Kit (Yeasen, 40706ES60). JC-1 assays were conducted in a 96-well plate, and luminescence was detected using a SpectraMax iD3 luminometer. For fluorescence detection, the excitation and emission wavelengths for JC-1 aggregates were set at 525/590 nm, while for JC-1 monomers, they were set at 490/530 nm.

### 4.15 Reverse transcription-quantitative polymerase chain reaction (RT-qPCR)

RNA was extracted from the anterior quarter of the larvae, or adult heads, or 293T cells using NucleoZol (MN, 740404. 200), following the manufacturer's instructions. The total RNA was then reverse-transcribed into cDNA using the PrimeScript™ RT Master Mix (Takara, RR036A-1, Japan). Quantitative PCR was carried out using the HieffTM qPCR SYBR® Green Master Mix (Yeasen, 11202ES08, China) on the Stratagene Mx3000P qPCR system. The primers utilized are listed below:

rp49 (Forward primer: CCACCAGTCGGATCGATATGC, Reverse primer: CTCTTGAGAACGCAGGCGACC);

Gp93 (Forward primer: CGGAGGTCAACCGCATGAT, Reverse primer: CAGAGCCAATAGGCGGATCTT);

Sod1 (Forward primer: GGACCGCACTTCAATCCGTA, Reverse primer: TGGAGTCGGTGATGTTGACC);

Cat (Forward primer: TTGGTTACTTTGAGGTGACCCA, Reverse primer: GGAGTGCGCTTCTTGACCTT);

β-actin (Forward primer: ATGAAGATCCTCACCGAGCG, Reverse primer: ACTCCATGCCCAGGAAGGAA);

rpr (Forward primer: TGGCATTCTACATACCCGATCA, Reverse primer: CCAGGAATCTCCACTGTGACT);

puc (Forward primer: CCGGCGGTCTACGATATAGAA, Reverse primer: TGGCAGGTATTTGCATGTACTT);

HSP90B1 (Forward primer: ATGGTCTGGCAACATGGAGAG, Reverse primer: GTTCAAACTGAGGCGAAGCAT);

SOD1 (Forward primer: AAAGATGGTGTGGCCGATGT, Reverse primer: CAAGCCAAACGACTTCCAGC).

### 4.16 Western blot

*Drosophila* larvae or 293T cells were rinsed with ice-cold PBS, followed by lysis using RIPA lysis buffer (Beyotime, P0013B, China) supplemented with a mixture of protease inhibitors (Yeasen, 20124ES03). To assess pJNK protein levels, a phosphatase inhibitor (Yeasen, 20109ES05) was also added to the lysis buffer. The lysates were then centrifuged at 15,000 rpm for 10 min at 4°C to pellet the cellular debris. Subsequently, proteins were fractionated by SDS-PAGE according to standard protocols. The antibodies used are indicated below: primary antibodies included Mouse anti-phospho-SAPK/JNK (Cell Signaling Technology, 9255S), Rabbit anti-cleaved caspase 3 (Cell Signaling Technology, 9664S), Rabbit anti-β-Actin (Cell Signaling Technology, 8457S), Mouse anti-Myc tag (Cell Signaling Technology, 2276S), and Rabbit anti-α-Tubulin (Cell Signaling Technology, 2125S). Secondary antibodies employed were Goat anti-rabbit IgG (Abways, AB0101) and Goat anti-mouse-IgG (Abways, AB0102).

### 4.17 Statistical analysis

Statistical analyses were performed using GraphPad Prism 9.0 software. The data are presented as the mean ± SEM for each experimental set. For comparisons between two groups, an unpaired *t*-test was employed. For experiments involving more than two groups with a single factor, ordinary one-way ANOVA was applied, followed by Dunnett's multiple-comparisons test for post hoc analysis. Survival data were analyzed using the Log-rank (Mantel-Cox) test. Ratio of ACV loss was analyzed using Fisher's exact test. Significance levels are indicated as ns: *P* > 0.05, ∗*P* < 0.05, ∗∗*P* < 0.01, ∗∗∗*P* < 0.001, ∗∗∗∗*P* < 0.0001.

## CRediT authorship contribution statement

**Meng Xu:** Conceptualization, Data curation, Formal analysis, Investigation, Methodology, Project administration, Software, Validation, Visualization, Writing – original draft, Writing – review & editing. **Wanzhen Li:** Data curation, Formal analysis, Methodology, Software, Visualization. **Ruihong Xu:** Data curation, Methodology, Software, Visualization. **Lixia Liu:** Data curation, Methodology, Software, Visualization. **Zhihan Wu:** Data curation, Methodology. **Wenzhe Li:** Methodology, Project administration, Funding acquisition. **Chao Ma:** Supervision. **Lei Xue:** Conceptualization, Data curation, Formal analysis, Funding acquisition, Investigation, Methodology, Project administration, Resources, Software, Supervision, Validation, Visualization, Writing – original draft, Writing – review & editing.

## Ethics approval and consent to participate

Not applicable.

## Consent for publication

All authors have read and agreed to the final version of the manuscript.

## Availability of data and materials

The data that support the finding of this study are available from the corresponding author upon reasonable request.

## Funding

This work was supported by the 10.13039/501100001809National Natural Science Foundation of China (31970536, 32370891), Shanghai Committee of Science and Technology (09DZ2260100, 19MC1910300), and the Fundamental Research Funds for the Central Universities (22120180549).

## Declaration of competing interest

The authors declare no competing interests.

## Abbreviations

ROS: reactive oxygen species; JNK: c-Jun N-terminal kinase; HSP90, heat shock protein 90; Gp93: Glycoprotein 93; GP96: Glycoprotein 96; Sod: superoxide dismutase; Cat, Catalase; Gtpx: glutathione peroxidase; Ask1: Apoptosis signal-regulating kinase 1; MAPKKK: mitogen-activated protein kinase kinase kinase; GSTP: Glutathione-S-transferase P; Bsk: Basket; JNKKs: JNK kinases; JNKKKs: JNK kinase kinases; Puc, puckered; PD, Parkinson's disease; AO: acridine orange; TNF, tumor necrosis factor; RNAi, RNA interference; ACV: anterior cross vein; A/P: anterior/posterior; C-cas3, cleaved caspase3; rpr, reaper; scrib, scribble; Hsp83, heat shock protein 83; UPR, unfolded protein response; PERK, pancreatic eIF-2α kinase; DHE: dihydroethidium; hSod1, human ortholog of Sod1; HSP70, heat shock protein 70; ER, endoplasmic reticulum; HSP90B1, heat shock protein 90 beta family member 1; HSPs, heat shock proteins; ALS, amyotrophic lateral sclerosis; annexin V-FITC, annexin V conjugated to fluorescein isothiocyanate; DCFH-DA, Dichlorodihydrofluorescein diacetate; RT-qPCR, reverse transcription-quantitative polymerase chain reaction.
