# Supplementary material for: Gp93 safeguards tissue homeostasis by preventing ROS-JNK-mediated apoptosis
Source: Redox Biol. 2025 Feb 8;81:103537. doi: 10.1016/j.redox.2025.103537 (PMC11875814; doi:10.1016/j.redox.2025.103537)
Supplement: Multimedia component 1 [file mmc1.pdf]

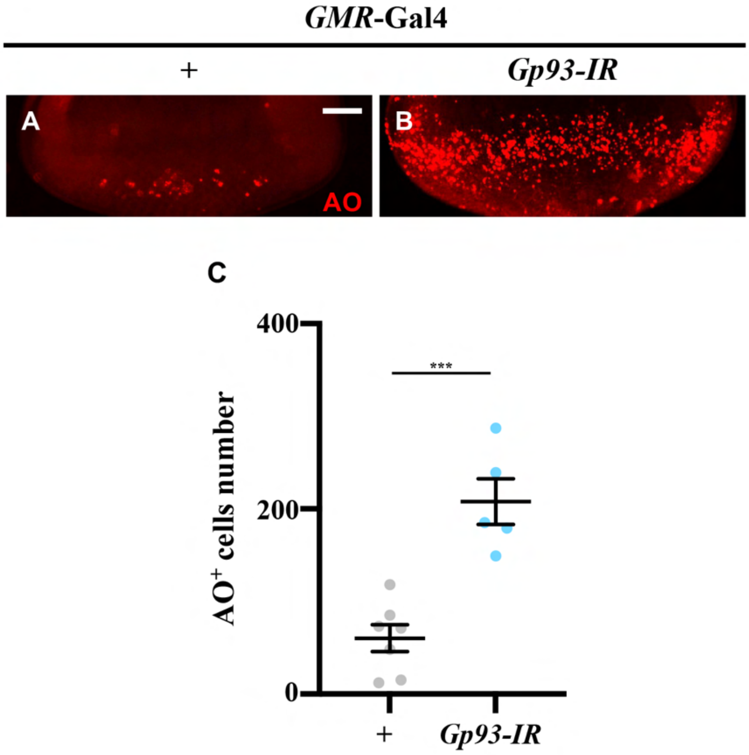

Figure S1. Knock down of *Gp93* induces cell death in eye-antennal discs.

Compared with *GMR*-Gal4 control (A), *GMR*>*Gp93-IR* induced excessive AO staining (B). (C) Statistical analysis of AO staining was performed using an unpaired *t*-test, ***P<0.001. Scale bar: 20 µm in A-B.

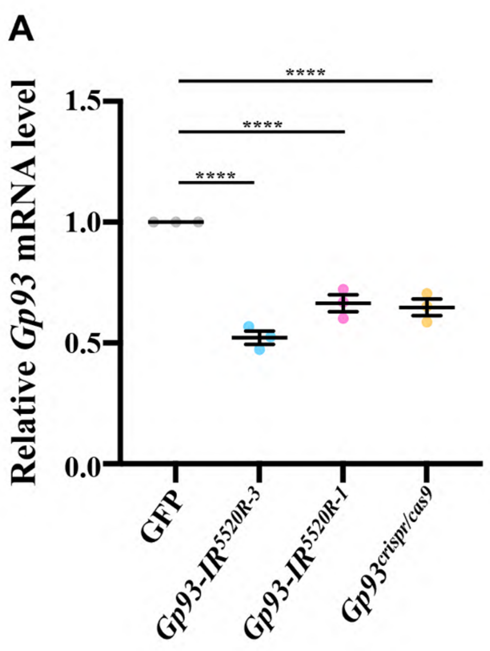

Figure S2. Characterization of *Gp93* knockdown and knockout strains.

(A) The knockdown or knockout of *Gp93* mediated by *act*-Gal4 decreases *Gp93* mRNA level compared with GFP-expressing control. RT-qPCR statistical analysis of *Gp93* mRNA was performed using a one-way ANOVA with post hoc Dunnett's multiple comparisons test, ****P<0.0001.

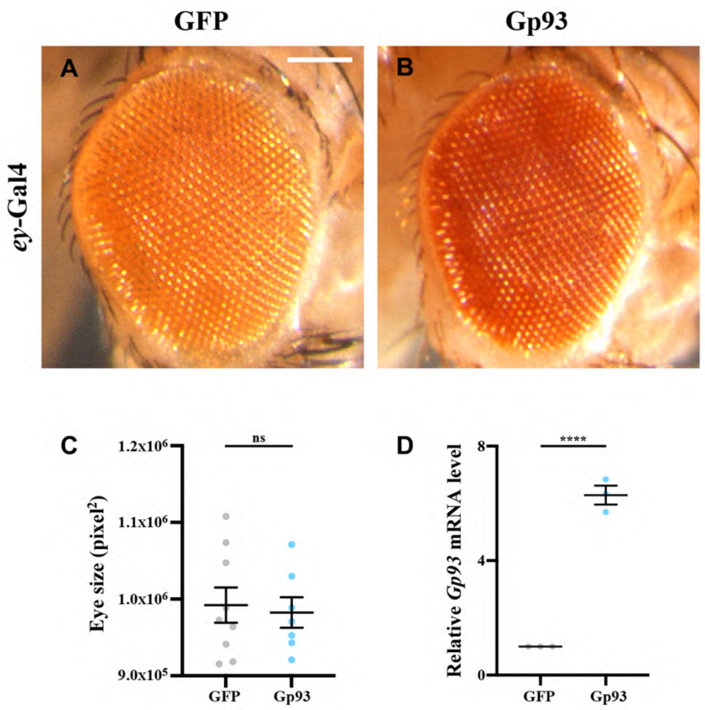

Figure S3. Gp93 overexpression does not affect eye size.

Compared with *ey*>GFP control (A), the eyes of *ey*>Gp93 remained the same size (B). (C) Statistical analysis of eye size was performed using an unpaired *t*-test, ns means no significance. (D) Statistical analysis of *Gp93* mRNA was performed using an unpaired *t*-test, ****P<0.0001. Scale bar: 100 µm in A-B.

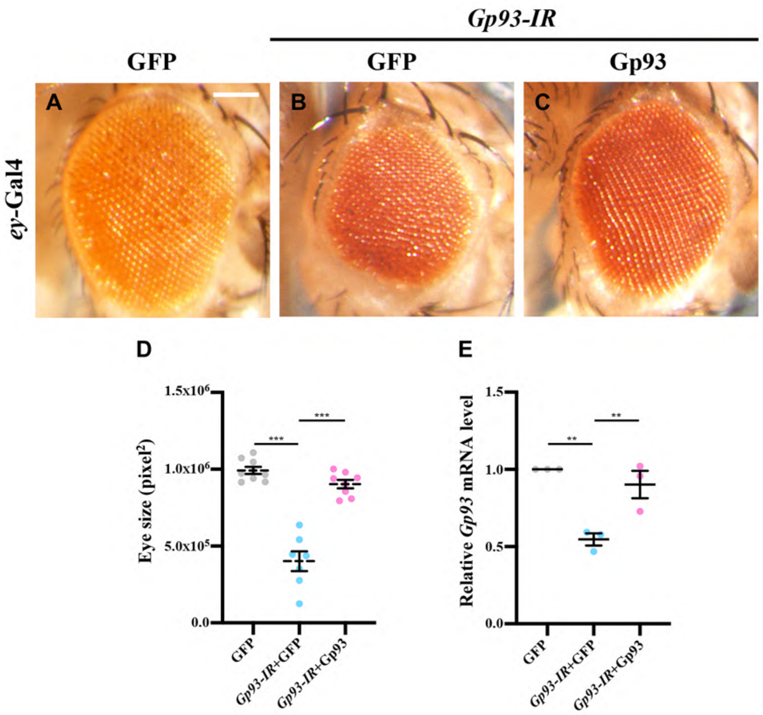

Figure S4. Gp93 overexpression suppresses *Gp93* depletion-induced smaller eyes. Compared with *ey*>GFP control (A), knockdown of *Gp93* induced smaller eyes (B), which could be suppressed by Gp93 overexpression (C). (D) Statistical analysis of eye size was performed using a one-way ANOVA with post hoc Dunnett's multiple comparisons test, ***P<0.001. (E) Compared with *act*>GFP control, *act*>*Gp93-IR*+GFP exhibited reduced *Gp93* mRNA level, which was restored by overexpression of Gp93. Statistical analysis of *Gp93* mRNA level was performed using a one-way ANOVA with post hoc Dunnett's multiple comparisons test, **P<0.01. Scale bar: 100 μm in A-C.

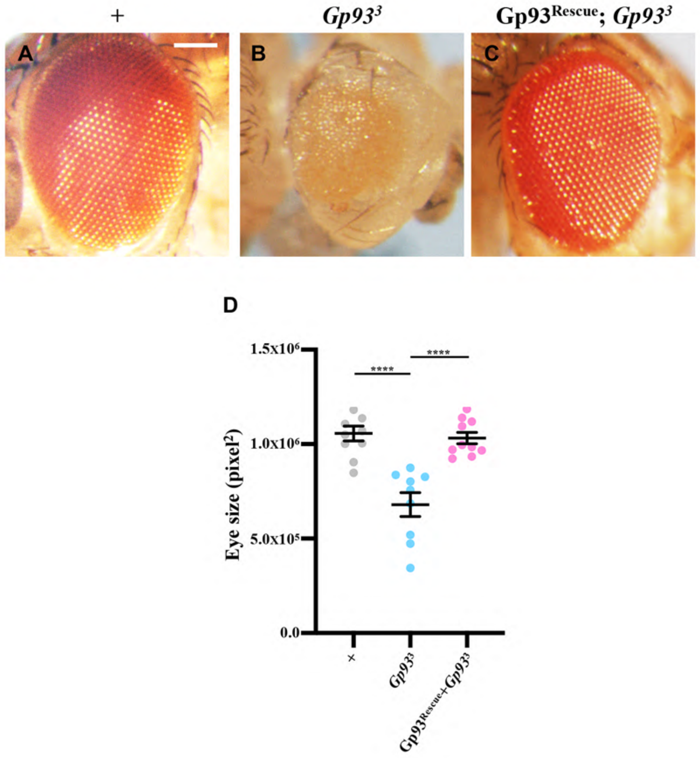

Figure S5. Loss of *Gp93* induces smaller eyes.

Adult *Drosophila* eyes were induced by EGUF (*Eyeless-Gal4-UAS-FLP*)/hid method, which combines *Gal4*/*UAS*, *FLP*/*FRT* systems and cell death gene *hid* expression to make *Gp93* homozygous mutant eyes. Compared with control (A), *Gp93* mutation induced smaller eyes (B), which could be reverted by genomic Gp93 expression (C). (D) Statistical analysis of eye size was performed using a one-way ANOVA with post hoc Dunnett's multiple comparisons test, ****P<0.0001. Scale bar: 100 µm in A-C.

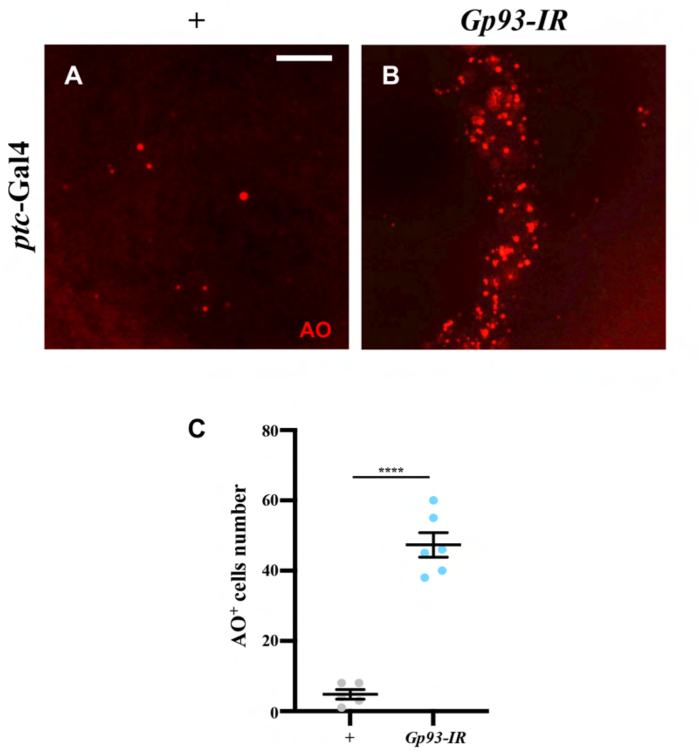

Figure S6. Knock down of Gp93 induces cell death in wing discs.

Compared with *ptc*-Gal4 control (A), *ptc*>*Gp93-IR* induced excessive AO staining (B). (C) Statistical analysis of AO staining was performed using an unpaired *t*-test, ****P<0.0001. Scale bar: 10 μm in A-B.

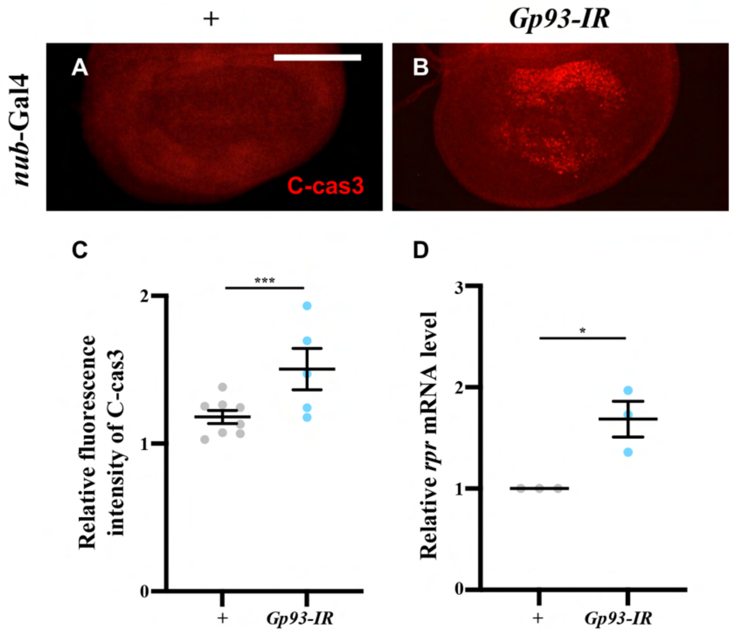

Figure S7. Knockdown of *Gp93* induces apoptosis.

Compared with *nub*-Gal4 control (A), *nub*>*Gp93-IR* induced cleavage of caspase 3 (C-cas3 (B). (C) Statistical analysis of C-cas3 staining was performed using an unpaired *t*-test, ***P<0.001. (D) Statistical analysis of *rpr* mRNA was performed using an unpaired *t*-test, *P<0.05. Scale bar: 100 μm in A-B.

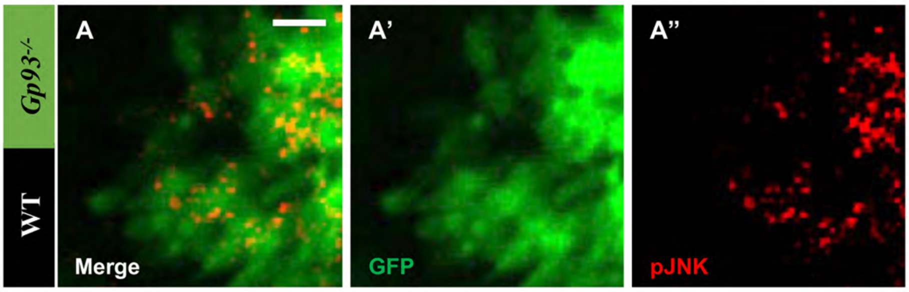

Figure S8. Loss of *Gp93* activates pJNK expression.

Gp93$^{3/3}$ mutant clones were induced in *y w*; *ey*-Flp *act*-Gal4 *UAS*-GFP/+; FRT82 *tub*-Gal80/FRT82 *Gp93*^{3} larval eye discs, stained for anti-pJNK antibody (red). In the absence of Gal80, *Gp93*^{3} mutant clones exhibited GFP expression. (A) Compared with wild type clones (without GFP), *Gp93*^{3} mutant clones (with GFP) displayed elevated pJNK staining. (A') *Gp93*^{3} mutant clones were labelled by GFP expression, whereas wild type clones showed no GFP expression. (A") Immunostaining for pJNK. Scale bar: 20 μm in A-A".

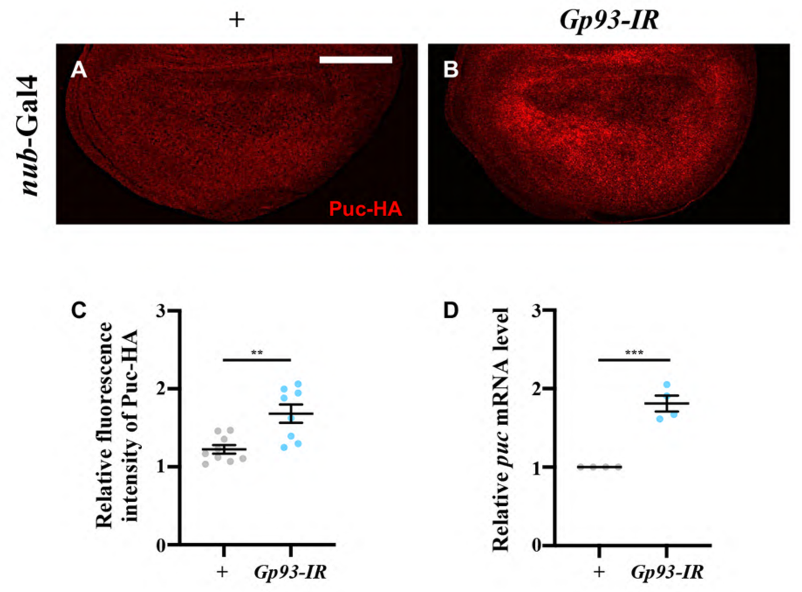

Figure S9. Knockdown of *Gp93* induces JNK pathway activation.

Compared with *nub*-Gal4 control (A), *nub*>*Gp93-IR* induced Puc-HA expression (B). (C) Statistical analysis of Puc-HA staining was performed using an unpaired *t*-test, $^{**}P<0.01$. (D) Compared with control, depletion of *Gp93* resulted in increased *puc* mRNA expression. RT-qPCR statistical analysis of *puc* mRNA expression was performed using an unpaired *t*-test, $^{***}P<0.001$. Scale bar: 50 μm in A-B.

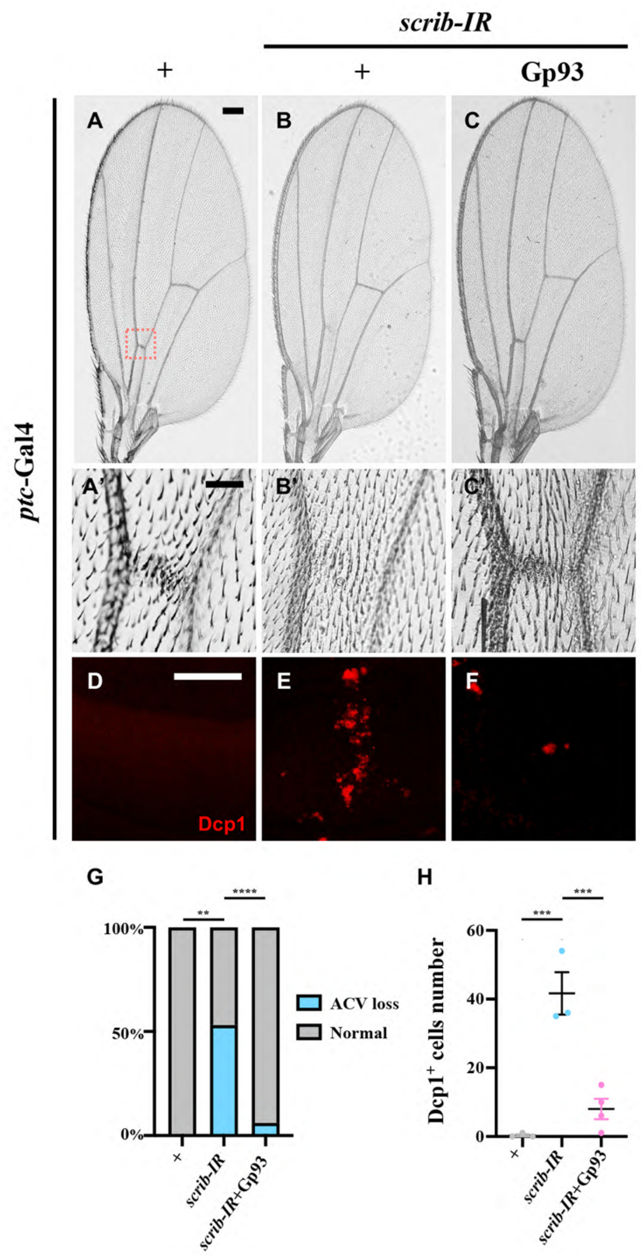

Figure S10. Gp93 overexpression suppresses cell death induced by *scrib*-IR.

Compared with the *ptc*-Gal4 control (A, A’, D), *scrib* depletion-induced ACV loss (B, B’) and cleaved Dcp1 staining (E) were suppressed by Gp93 overexpression (C, C’, F). Red dashed lines outline the region of ACV. (A’-C’) Magnification of ACV region. Statistical analysis of ACV loss was performed using Fisher’s exact test (G). Statistical analysis of Dcp1 staining (H) was performed using a one-way ANOVA with post hoc

Dunnett's multiple comparisons test, $**P<0.01$, $***P<0.001$ and $****P<0.0001$. Scale bar: 100 μm in A-C, 20 μm in A'-C' and D-F.

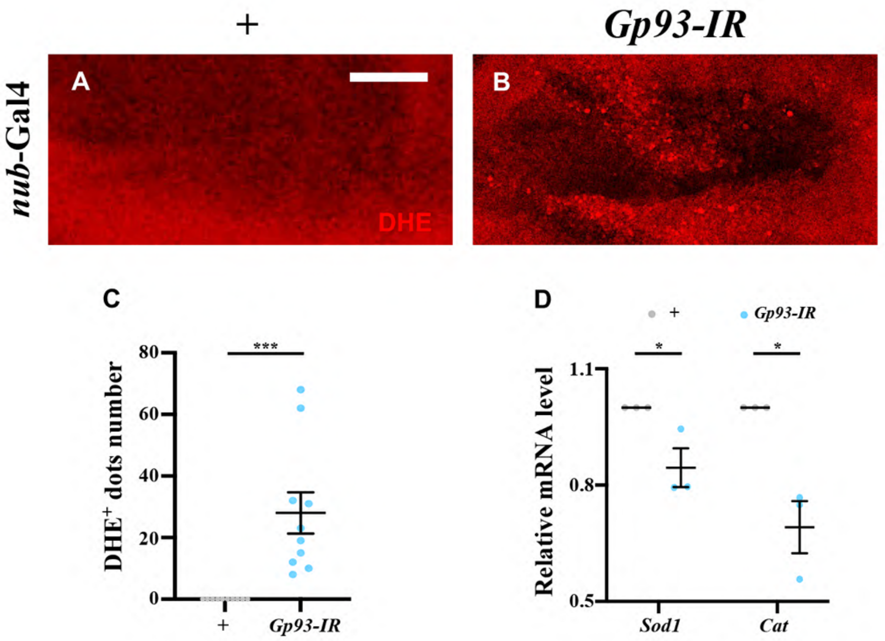

Figure S11. Loss of *Gp93* induces ROS generation.

(A) Compared with *nub*-Gal4 control, (B) *nub*>*Gp93-IR* induced DHE staining. (C) Statistical analysis of DHE staining is shown with an unpaired *t*-test, ***P<0.001. (D) Compared with control, knockdown of *Gp93* led to reduced *Sod1* or *Cat* mRNA expression. RT-qPCR statistical analysis of *Sod1* and *Cat* mRNA is shown: unpaired *t*-test, *P<0.05. Scale bar: 25 μm in A-B.

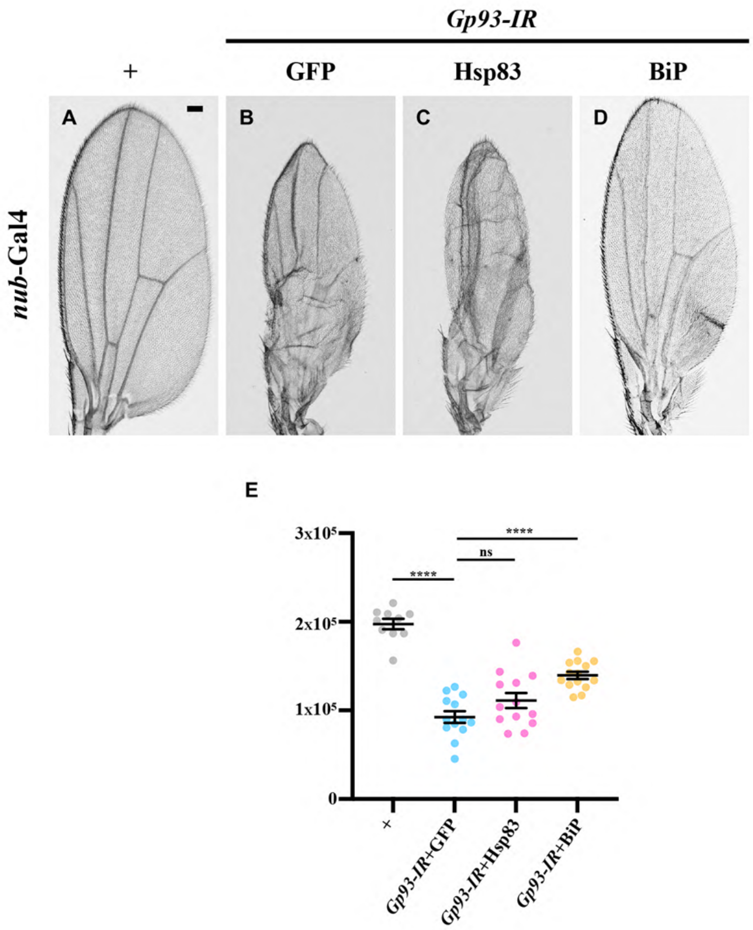

Figure S12. BiP suppresses *Gp93* knockdown-induced wing size reduction.

Compared with *nub*-Gal4 control (A), *Gp93* knockdown-induced smaller wings (B) were not suppressed by expressing Hsp83 (C), but suppressed by expressing BiP (D). (E) Statistical analysis of wing size was performed using a one-way ANOVA with post hoc Dunnett's multiple comparisons test, $^{****}P<0.0001$ and ns means no significance. Scale bar: 100 μm in A-D.

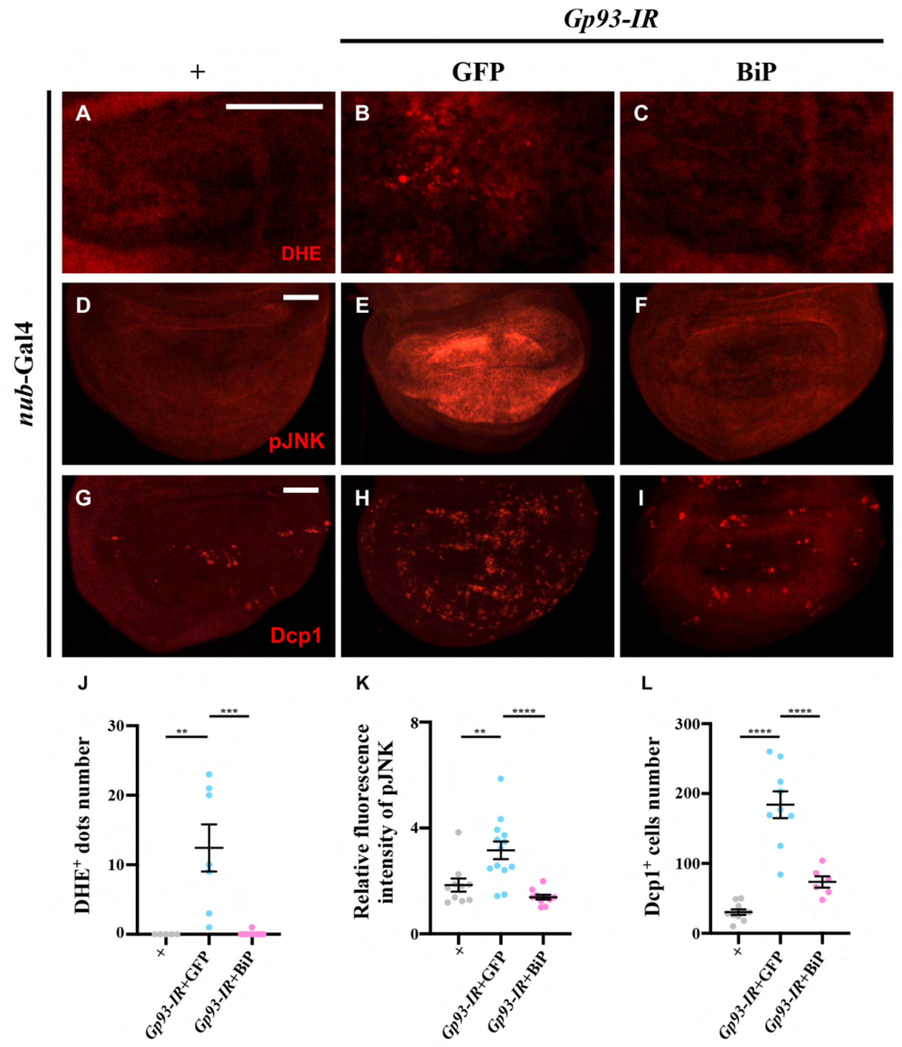

Figure S13. BiP suppresses *Gp93* depletion-induced ROS-JNK-mediated apoptosis.

Compared with *nub*-Gal4 control (A, D and G), *Gp93* depletion-induced ROS, JNK activation and apoptosis (B, E and H) were suppressed by BiP overexpression (C, F and I). Statistical analysis of DHE staining (J), pJNK staining (K) and Dcp1 staining (L) were performed using a one-way ANOVA with post hoc Dunnett's multiple comparisons test. **P<0.01 and ****P<0.0001. Scale bar: 50 μm in A-I.

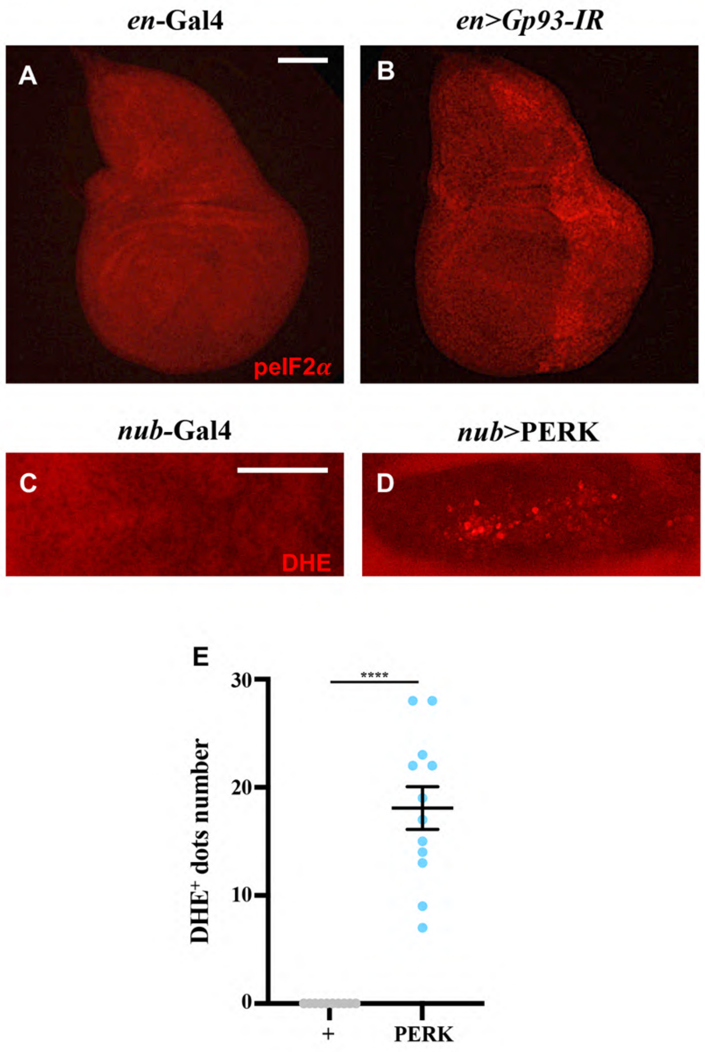

Figure S14. PERK overexpression induces ROS.

Compared with *en*-Gal4 control (A), depletion of *Gp93* induced eIF2α phosphorylation (B). (C) Compared with *nub*-Gal4 control, PERK overexpression induced DHE staining. (E) Statistical analysis of DHE staining is shown: unpaired *t*-test, *****P*<0.0001. Scale bar: 50 µm in A-D.

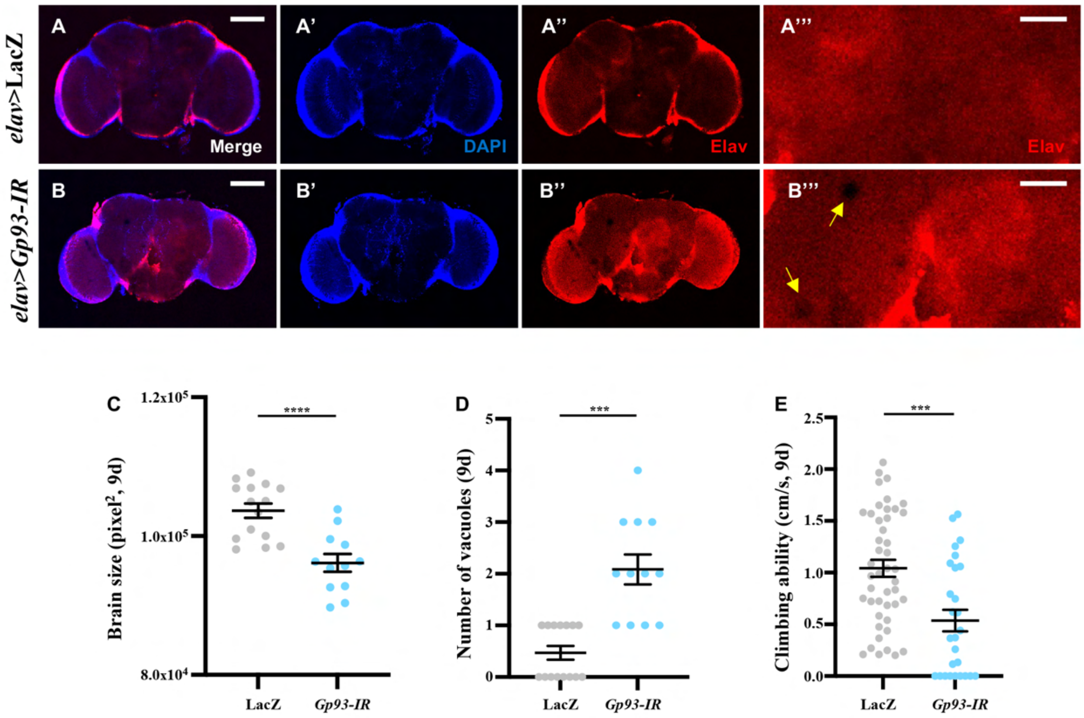

Figure S15. Knockdown of *Gp93* induces neurodegeneration.

Compared with *elav*>LacZ control (A), *elav*>*Gp93-IR* induced smaller brains and vacuoles formation (B). (A'-B') DAPI staining of brains. (A''-B'') Elav staining of brains. (A'''-B''') Magnification of brains, the yellow arrow points to vacuole. Statistical analyses of brains size (C) and vacuoles number (D) are shown: unpaired *t*-test, ****P*<0.001 and *****P*<0.0001. (E) The average speed of 9-day-old flies climbing up from the bottom of the tube within 5 seconds was statistically measured. Comparing with *elav*>LacZ control, climbing speed of *elav*>*Gp93-IR* was reduced. Statistical analysis of climbing ability was performed using an unpaired *t*-test, ****P*<0.001. Scale bar: 100 µm in A-A'' and B-B'', 50 µm in A''' and B'''.

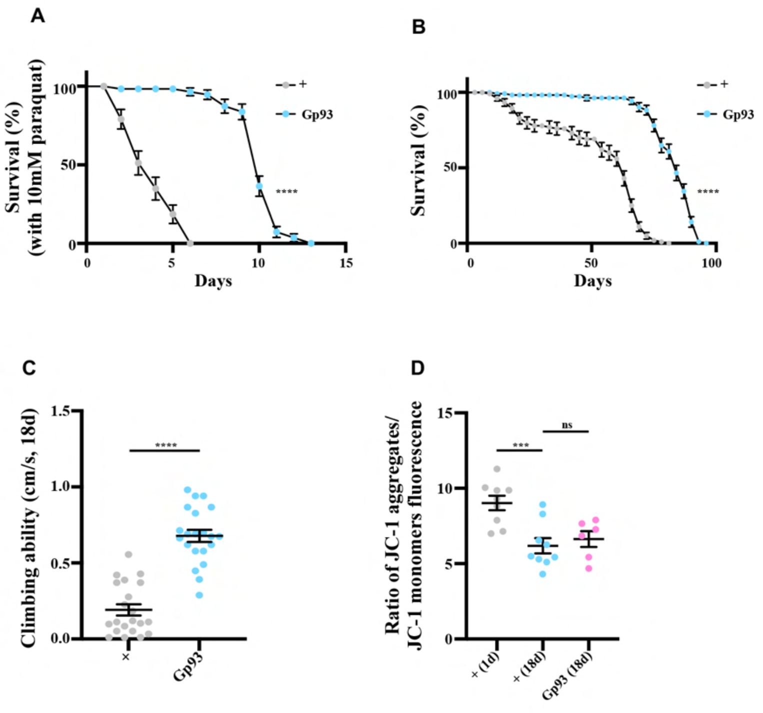

Figure S16. Gp93 promotes lifespan and climbing ability.

(A) Treated with 10 mM paraquat, *elav*>Gp93 flies had prolonged lifespan compared with control. Survival analysis of flies is shown: Log-rank (Mantel-Cox) test, ****P<0.0001. (B) Compared with control, *elav*>Gp93 flies had prolonged lifespan compared with control. Survival analysis of flies is shown: Log-rank (Mantel-Cox) test, ****P<0.0001. (C) The average speed of 18-day-old flies climbing up from the bottom of the tube within 5 seconds was statistically measured. Compared with control, crawling speed of *elav*>Gp93 was enhanced. Statistical analysis of climbing ability was performed using an unpaired *t*-test, ****P<0.0001. (D) The fluorescence of JC-1 aggregates indicates a higher mitochondrial membrane potential, generally signifying the presence of healthy mitochondria. Conversely, the fluorescence of JC-1 monomers suggests a lower mitochondrial membrane potential, typically indicating unhealthy

mitochondria. The ratio of JC-1 aggregate fluorescence to JC-1 monomer fluorescence reflects alterations in mitochondrial membrane potential. When compared with the control group (1-day-old flies), brains of 18-day-old flies exhibited a diminished mitochondrial membrane potential, a condition that was not alleviated by the overexpression of Gp93. Statistical analysis of JC-1 fluorescence was performed using a one-way ANOVA with post hoc Dunnett's multiple comparisons test, ***$P<0.001$ and ns means no significance.

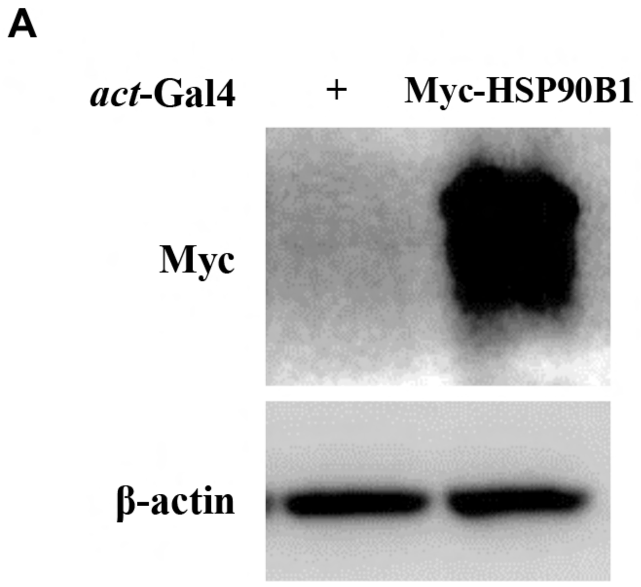

Figure S17. Western blot analysis of HSP90B1 expression in transgenic flies.

In comparison to the control group, the level of Myc-HSP90B1 protein was significantly elevated in *act*>HSP90B1 larvae.

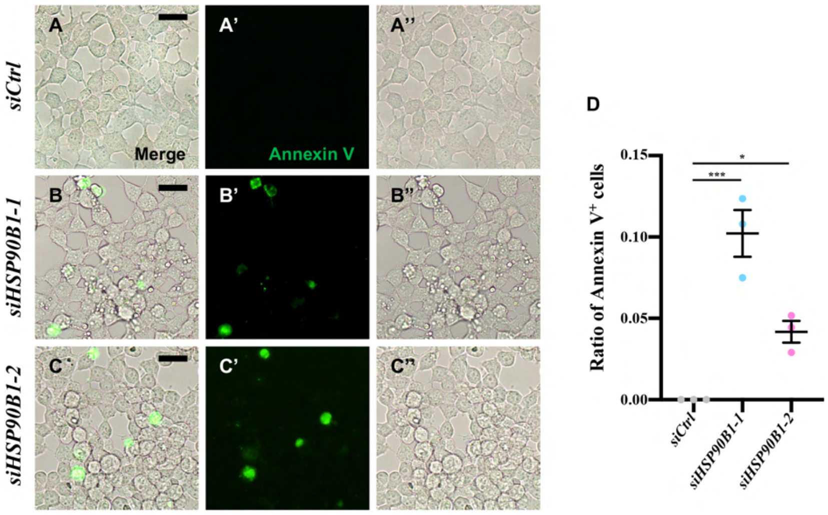

Figure S18. Depletion of *HSP90B1* induces apoptosis.

Compared with control (A-A''), knockdown of *HSP90B1* in human 293T cells by two independent RNAi induced Annexin V staining (B-B'', C-C''). (D) Statistical analysis of Annexin V staining was performed using a one-way ANOVA with post hoc Dunnett's multiple comparisons test. *P<0.05 and ***P<0.001. Scale bar: 100 μm in A-C''.
